# Live imaging of the *Drosophila* ovarian niche shows spectrosome and centrosome dynamics during asymmetric germline stem cell division

**DOI:** 10.1242/dev.199716

**Published:** 2021-09-17

**Authors:** Gema Villa-Fombuena, María Lobo-Pecellín, Miriam Marín-Menguiano, Patricia Rojas-Ríos, Acaimo González-Reyes

**Affiliations:** Centro Andaluz de Biología del Desarrollo, CSIC/Universidad Pablo de Olavide/JA, Carretera de Utrera km 1, 41013 Sevilla, Spain

**Keywords:** *Drosophila* oogenesis, Germline stem cells, Spectrosome, Centrosome separation, Stem cell division, Live imaging

## Abstract

*Drosophila* female germline stem cells (GSCs) are found inside the cellular niche at the tip of the ovary. They undergo asymmetric divisions to renew the stem cell lineage and to produce sibling cystoblasts that will in turn enter differentiation. GSCs and cystoblasts contain spectrosomes, membranous structures essential for orientation of the mitotic spindle and that, particularly in GSCs, change shape depending on the cell cycle phase. Using live imaging and a fusion protein of GFP and the spectrosome component Par-1, we follow the complete spectrosome cycle throughout GSC division and quantify the relative duration of the different spectrosome shapes. We also determine that the Par-1 kinase shuttles between the spectrosome and the cytoplasm during mitosis and observe the continuous addition of new material to the GSC and cystoblast spectrosomes. Next, we use the Fly-FUCCI tool to define, in live and fixed tissues, that GSCs have a shorter G1 compared with the G2 phase. The observation of centrosomes in dividing GSCs allowed us to determine that centrosomes separate very early in G1, before centriole duplication. Furthermore, we show that the anterior centrosome associates with the spectrosome only during mitosis and that, upon mitotic spindle assembly, it translocates to the cell cortex, where it remains anchored until centrosome separation. Finally, we demonstrate that the asymmetric division of GSCs is not an intrinsic property of these cells, as the spectrosome of GSC-like cells located outside of the niche can divide symmetrically. Thus, GSCs display unique properties during division, a behaviour influenced by the surrounding niche.

## Abstract (precis)

**Summary:** Imaging of live *Drosophila* germline stem cells in the ovarian niche reveals their asymmetric division and centrosome behaviour, whereas tumorous stem cells divide symmetrically.

## INTRODUCTION

Germ cells are the gamete precursors and, therefore, key components of sexual reproduction. Normally set aside from somatic lineages during early embryogenesis, the strategies to supply functional gametes often require the stem cell system. Among these, the reproductive organs of the fruit fly *Drosophila melanogaster* develop niches for germline stem cells (GSCs) and somatic stem cells, which are ultimately responsible for the generation of new gametes.

**Fig. 1.**
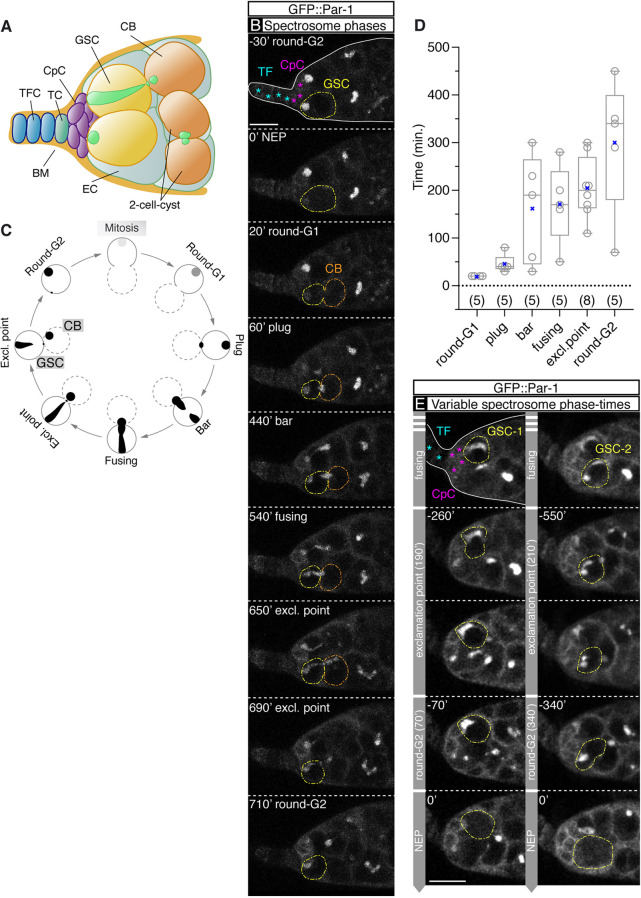
**Live dynamics of the GSC spectrosome cycle.** (A) Drawing of a germline stem cell (GSC) niche showing terminal filament cells (TFCs), the transition cell (TC), cap cells (CpCs), escort cells (ECs), the basement membrane (BM), two GSCs, a cystoblast (CB) and a two-cell cyst (spectrosomes are in green). (B) GSC spectrosome morphology before and after mitosis visualised with GFP::Par-1. Nuclear envelope permeation (NEP) sets the t=0′ point. (C) Morphologies adopted by the spectrosome during the GSC cycle. Dotted lines represent the future CB. The spectrosomes in Mitosis and early in G1 are depicted in grey to represent the release of GFP::Par-1. (D) Quantification of the duration of each spectrosome morphology in 14 germaria. The mean (cross) and median (line across box) values are shown. (*n*) sample size. (E) Time-lapse stills of a *w;; pUbi-GFP:: par-1* germarium showing two GSCs transitioning from ‘fusing’ to ‘exclamation point’ to ‘round-G2’ to ‘NEP’. The duration of the ‘exclamation point’ phases are similar, but the assessed ‘round-G2’ phases are markedly different (NEP, t=0′). The stills are maximum projections of 3-8 *z*-planes, each 1 μm apart. Times correspond to the initiation of the ‘exclamation point’, ‘round-G2’ and ‘NEP’ phases. Scale bars: 10 μm. Related to Fig. S1. Panel B related to Movie 1.

*Drosophila* ovaries are composed of 16-18 ovarioles that sustain egg chamber development to produce mature eggs during the lifespan of the animal. Egg chambers are generated in the germarium, a tapered structure at the anterior end of the ovariole that hosts a limited number of GSCs. The GSC niche contains three cell types of somatic origin: terminal filament cells (TFCs), cap cells (CpCs) and anterior escort cells (ECs; Fig. 1A). These niche cells provide GSCs with signals and physical support to prevent entry into differentiation (Eliazer and Buszczak, 2011). The organisation of the ovarian niche is very well established and includes a specialised extracellular matrix, a terminal filament (TF) of 8-10 cells, a rosette of 6-8 CpCs connected to the TF via the ‘transition cell’ (TC) and 2-3 ECs placed in close contact with the CpCs (Díaz-Torres et al., 2021; Panchal et al., 2017; Wang and Page-McCaw, 2018). This microenvironment orchestrates short-range signalling and provides physical space to maintain 2-4 GSCs per niche. In addition, it also integrates systemic factors such as insulin signalling, nutritional state, steroid hormones and age, among others (Drummond-Barbosa, 2019; Eliazer and Buszczak, 2011), to help maintain a functional niche. GSCs normally divide asymmetrically (i.e. each daughter cell acquires a different fate) to produce a lineage-renewing GSC and a sister cell termed a cystoblast (CB), which is destined for differentiation. GSCs and CBs possess an intracellular organelle called a spectrosome that is highly enriched in small vesicles and associated proteins such as the serine-threonine kinase Par-1 and the membrane skeletal component Hu-li tai shao (Hts), homologue of mammalian adducin (Huynh et al., 2001; Lin et al., 1994; Yue and Spradling, 1992; Lighthouse et al., 2008). The shape of the GSC spectrosome varies throughout the cell cycle and it can be used as a morphological marker to help distinguish between the GSC Gap 1 (G1), Synthesis (S), Gap 2 (G2) and Mitosis (M) phases (Ables and Drummond-Barbosa, 2013). Female GSCs undergo mitosis without proper nuclear envelope breakdown, as the nuclear lamina remains intact during division, albeit the mitotic nuclear envelope becomes permeable (Duan et al., 2021). Later in oogenesis, the CB spectrosome grows into a branched figure called a fusome, characteristic of differentiating germline cysts (de Cuevas and Spradling, 1998; Ong and Tan, 2010).

Communication between CpCs, ECs and GSCs permits proper GSC proliferation and prevents their differentiation. A number of signalling cascades are active in the niche, including the Dpp (*decapentaplegic*, which encodes the BMP2/4 orthologue in *Drosophila*) pathway. Dpp is produced in CpCs and ECs and is received in the GSCs via its type I (Thickveins and Saxophone) and type II (Punt) receptors (López-Onieva et al., 2008; Rojas-Ríos et al., 2012; Wang et al., 2008; Xie and Spradling, 1998). Depletion of Dpp signalling in the niche induces stem cell differentiation and loss of the GSC lineage. Conversely, increased Dpp signalling generates tumorous masses of GSC-like cells and prevents formation of fusome structures, as seen after overexpression of *dpp* or of an activated form of the Thickveins receptor (Xie and Spradling, 1998; Casanueva and Ferguson, 2004).

We define experimental conditions that allow prolonged observations of *ex vivo*-cultured germaria to characterise different aspects of GSC division. We quantify the duration of different cell cycle phases (M in greater detail), establish the correlation between spectrosome morphology and GSC cell cycle phase, determine the behaviour of the GSC centrosomes before mitosis and report that the GSC centrosome separates without centriole duplication. Finally, we analyse the proliferation of GSC-like tumours and describe that GSCs divide symmetrically (i.e. both daughter cells inherit similarly sized spectrosomes) in these tumours.

## RESULTS

### The dynamic changes in spectrosome morphology in living GSCs

The 2-4 GSCs present in a germarial niche are easily recognisable by their location at the base of the CpC rosette and by the presence of a prominent spectrosome. The female GSC spectrosome undergoes remarkable morphological changes during the cell cycle and several shapes have been defined in fixed tissues throughout interphase and mitosis (Ables and Drummond-Barbosa, 2013; de Cuevas and Spradling, 1998; Deng and Lin, 1997; Hsu et al., 2008; LaFever et al., 2010). To describe accurately the dynamics of spectrosome morphologies, we have filmed for several hours live germaria expressing ubiquitously a GFP::Par-1 fusion protein that decorates spectrosomes and fusomes, in addition to labelling cell membranes (Fig. 1B). Par-1 is a component of the spectrosome and fusome and colocalises with Hts, another well-characterised spectrosome/fusome marker (Cox et al., 2001; Huynh et al., 2001; Lin et al., 1994; Vaccari and Ephrussi, 2002). Our culturing conditions, which required the use of a tissue adhesive and bottom-glass plates, allowed the imaging of live tissue for at least 16 h without obvious deleterious defects in the niche (Fig. S1A). The general signal present in the GFP::Par-1-expressing cells allowed us to discriminate entry into mitosis, as the nucleoplasm was filled with GFP::Par-1 signal once the nuclear envelope became permeable in early prophase (Fig. 1B; Movies 1 and 2). Throughout this work and in those examples in which we could distinguish nuclear envelope permeation (NEP), this event set the time to 0 min (t=0′). We filmed 23 GSCs from 11 germaria that underwent mitosis.

We confirm that the GSC spectrosome is asymmetrically partitioned between the two daughter cells and define five distinct spectrosome morphologies that extended during a complete GSC cell cycle. Right after mitosis, the spectrosome displayed a ‘round’ morphology and it was placed at the anterior margin of the cell, abutting the CpC rosette. Next, new spectrosome material appeared filling the cytokinetic ring that connected the daughter GSC and its sibling, the prospective CB, defining – together with the original, anteriorly placed spectrosome – the ‘plug’ morphology. As the ring material appeared soon after mitosis, and as the anterior, round portion of the ‘plug’ spectrosome appeared to be smaller than the one before mitosis, we suggest that the spectrosome material filling the ring canal at the ‘plug’ stage comes from the anterior spectrosome. Subsequently, the newly-formed plug and the anterior portion of the spectrosome incorporated new spectrosome material and projected towards each other, thus defining the ‘bar’ shape. Once both portions connected with each other, the spectrosome extended the entire length of the GSC, from the anterior margin of the cell abutting the cap cells to the connection with the forming CB. This spectrosome morphology was classified as ‘fusing’. Afterwards, and as cytokinesis was completed, the spectrosome material was strangled at the cytokinetic ring, giving rise to the ‘exclamation point’ morphology. Finally, the severed, elongated spectrosome inside the GSC recoiled to its anterior position, becoming round again (Fig. 1B; Movie 1). Thus, our live imaging confirmed previous reports (LaFever et al., 2010) and determined that spectrosomes in living GSCs cycled from a ‘round’ appearance right after mitosis (which we termed ‘round-G1’ in correlation with the G1 cell cycle phase), to a ‘plug’ morphology, in which the spectrosome is divided into two fragments and during which new material began to merge onto the equatorial piece, to the ‘bar’ and ‘fusing’ shapes that resulted from the growth of both spectrosome fragments, to the ‘exclamation point’ figure observed upon cytokinesis and to a ‘round-G2’ morphology found in GSCs before M phase (Fig. 1C; Fig. S1B).

To quantify the duration of the different phases, we analysed in detail over 205 h of long-duration movies (up to 16 h each). Upon closer analysis of 27 GSCs from 15 *GFP::Par-1* germaria, we found several examples of GSCs in which the spectrosomes transitioned, in the same movie, between three different phases (i.e. from ‘plug’ to ‘bar’ to ‘fusing’), allowing us to determine precisely the duration of the intermediate phase (in the above case, ‘bar’; Fig. 1D). The time resolution of these observed phases was limited by the settings of our confocal movies, with 10-min time intervals to prevent excessive bleaching of the signal. All the scored ‘round-G1’ took place in two time points, thus the average duration for this phase was 20 min (*n*=5). The rest of the phases provided less uniform values: ‘plug’ lasted between 30-80 min (mean±s.e.m., 46±8.72, *n*=5), ‘bar’ between 30-300 min (162±51.13, *n*=5), ‘fusing’ between 50-280 min (172.5±4.80, *n*=5), ‘exclamation point’ between 110-300 min (205±22.68, *n*=8) and ‘round-G2’ lasted for 70-450 min (300±63.09, *n*=5; Fig. 1D). Thus, in good agreement with published data showing that GSCs from germaria cultured in insulin-supplemented media divided on average once every 12-14 h (Morris and Spradling, 2011), we determined that a GSC division cycle takes on average ∼15.5 h. Finally, although the dispersion in the durations of a given spectrosome morphology could be considerable, we believe this spread in time values represented true variability of the process. We managed to identify several examples of GSCs belonging to the same germarium that went through similar phase transitions in the same movie and that gave variable spectrosome phase durations. For example, neighbouring GSCs gave values of 40 min and 80 min for the ‘plug’ phase, 30 min and 230 min or 190 min and 300 min for ‘bar’, 50 min and 280 min or 160 min and 200 min for ‘fusing’, 210 min and 290 min for ‘exclamation point’, and 70 min and 450 min or 290 min and 450 min for ‘round-G2’ (Fig. 1E).

### The Par-1 kinase is released from spectrosomes and fusomes during mitosis

**Fig. 2.**
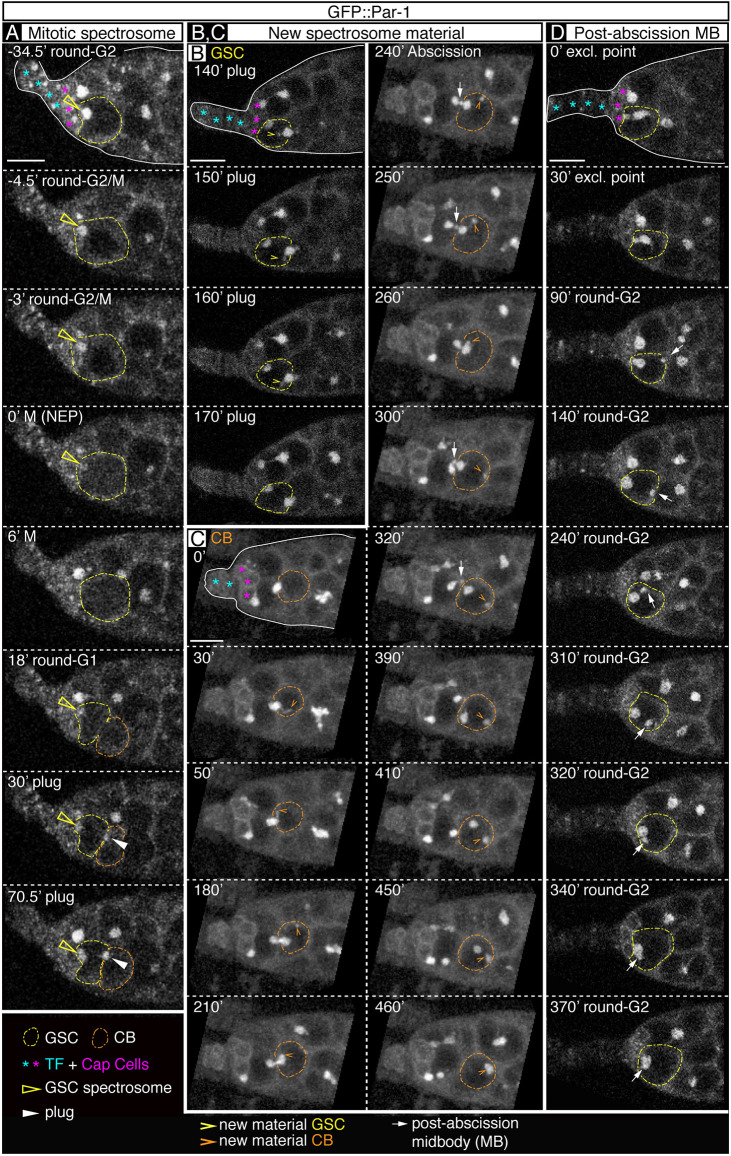
**GFP::Par-1 is released from the GSC spectrosome during mitosis.** (A-D) Addition of new material during spectrosome growth. The germline stem cell (GSC) inherits the post-abscission midbody. (A) GFP::Par-1 is released from the mitotic spectrosome. Soon after mitosis, GFP::Par-1 re-localises to both the round spectrosome and the cytokinetic plug. (B,C) New material is transported to, and accumulated on, GSC (B) and cystoblast (CB; C) spectrosomes. Spectrosome abscission and the resulting midbody are shown. (D) The post-abscission midbody is inherited by the GSC and eventually fuses with the ‘round-G2’ spectrosome. NEP, nuclear envelope permeation. Scale bars: 10 μm. Related to Figs S1-S3. Panel A related to Movies 2 and 3, panel B to Movie 4, panel C to Movie 6 and panel D to Movie 7.

We observed that the vivid GFP::Par-1 signal of ‘round-G2’ spectrosomes faded in G2-M-G1 transitions, whereas its cytoplasmic staining increased. Imaging live *GFP::par-1* germaria with shorter time intervals (every 1.5 min), we determined that bright, clearly visible ‘round-G2’ spectrosomes became progressively devoid of the GFP signal as early as 10 min before NEP (t=−10′). Upon NEP (t=0′) the GFP signal filled the nuclear space, indicating that the GFP::Par-1 protein was released from the spectrosome. After mitosis and the reformation of the nuclear envelope, the spectrosome signal was recovered in all cases analysed and the ‘round-G1’ spectrosome was clearly visible some 20 min after NEP (Fig. 2A; Movie 2; Fig. S1C). This behaviour is not exclusive of female GSCs, as we have also observed the fading of the GFP::Par-1 signal in the spectrosome of dividing CBs and in the fusome of mitotic two-, four- and eight-cell cysts (Movie 3 shows a four-cell cyst division). As mitotic spectrosomes maintained strong anti-Hts staining in fixed tissue (Fig. S1D), as previously reported also for α-spectrin (de Cuevas and Spradling, 1998; Deng and Lin, 1997; Lin and Spradling, 1995), the disappearance of GFP::Par-1 from the spectrosome of dividing GSCs is most likely a particularity of this kinase and not an indication of spectrosome disassembly.

### New material incorporates into the growing GSC and CB spectrosomes

The dynamics of the spectrosome cycle suggested that both fragments of spectrosome material seen after GSC division in the ‘plug’ phase increased in size to account for the ‘bar’, ‘fusing’ and ‘exclamation point’ morphologies. We thus focused on ‘plug’ and ‘bar’ figures and looked for *de novo* addition of GFP::Par-1 material to the anterior spectrosome and to the middle fragment filling the cytokinetic ring. In all of our movies, we could detect frequent GFP-positive threads and vesicles in the GSC/CB pairs that showed a directed movement towards the middle fragment, strongly suggesting that equatorial spectrosome growth depends on the incorporation of *de novo* synthesised material (Fig. 2B,C; Movies 4 and 6). In the anterior GSC, the new Par-1-positive substance was added in the form of elongated threads and they were less abundant and smaller than those observed in the CB side of the equatorial plug. In the latter cell, the material incorporating into the plug appeared to originate from the posterior half and was always associated with the cell membrane, either in the form of threads or as small vesicles. The anterior ‘round-G2’ spectrosome incorporated little new material compared with the growth observed in the ‘plug’, ‘bar’ and ‘fusing’ phases. However, we could detect traces of new GFP::Par-1 matter merging onto ‘round-G2’ spectrosomes until a few moments before mitosis (Fig. S2A; Movie 5).

Of interest, we also noticed at later stages of CB maturation the generation of a large GFP::Par-1 globule that remained located at the posterior pole. Upon complete abscission of the GSC spectrosome at the ‘exclamation point’ phase and the release of the CB spectrosome from the cytokinetic furrow, the latter moved towards the posterior pole, where it eventually fused with the newly generated, posteriorly-placed GFP::Par-1 sphere. The resulting spectrosome almost doubled in size that of the CB (Fig. 2C; Movie 6). Finally, to test whether the above findings reflected a general mechanism of spectrosome growth, we looked at the distribution of the spectrosome component Hts in fixed GSC and CBs and found accumulations of Hts-positive structures in these cells consistent with new material being incorporated to the growing spectrosomes in a similar fashion to Par-1 (Fig. S2B). Our results thus indicate that new material containing Par-1 and likely other components such as Hts are added mainly to the equatorial spectrosome both in the GSC and in the prospective CB, primarily during the ‘plug’, ‘bar’ and ‘fusing’ phases. In addition, CB spectrosomes result from the fusion of its original, cytokinetic ring-associated material and a posteriorly located mass of new assembled material.

### The post-abscission midbody eventually fuses with the apical spectrosome

In contrast to CBs and differentiating germline cysts, which block cytokinesis after division, GSCs undergo complete cytokinesis in G2 (de Cuevas and Spradling, 1998; Matias et al., 2015). Cytokinesis implies the specification of the cleavage plane and the ingression of the contractile actomyosin ring, bracing a microtubule-rich proteinaceous structure known as the midbody (MB). The final step of cytokinesis is abscission, upon which the plasma membrane of both daughter cells physically separates and the post-abscission MB is generated. In the case of the *Drosophila* germline, it has been reported that male GSCs do not inherit the post-abscission MB, whereas female GSCs do (Matias et al., 2015; Salzmann et al., 2014). We performed a detailed analysis of post-abscission MB behaviour in our high-resolution long-duration movies using the GFP::Par-1 fusion. The formation of the MB can be discerned with the GFP::Par-1 marker as the contraction of the ring strangles the ‘fusing’ spectrosome traversing the cytokinetic ring to give rise to the ‘exclamation point’ figure (Fig. 1B). Upon completion of the GSC/CB abscission, the newly formed post-abscission MB remained associated to the GSC plasma membrane and drifted for several hours until it finally fused to the anterior ‘round-G2’ spectrosome (Fig. 2D; Movie 7). We observed this behaviour in all seven GSC divisions in which we could follow the release of the post-abscission MB from the equatorial side of the GSC/CB pair until its fusion with the spectrosome. Our results thus confirmed previous findings and raised the question of a possible role for the post-abscission MB on female GSC biology, as post-abscission MBs can act as signalling platforms to regulate cell polarity, tumorigenesis and stemness (reviewed by Peterman and Prekeris, 2019).

### Exceptions to the canonical spectrosome cycle: posterior GSC spectrosomes

We observed a number of GSCs, the spectrosomes of which deviated from the canonical cycle described above. Of the 23 GSCs that underwent mitosis in our long-duration movies, 11 lasted long enough as to follow the spectrosome for several hours after mitosis. The apical spectrosome detached from the GSC-CpC interface and moved to the cell posterior in four cases, leaving a ‘scar’ of spectrosome material at the apical side (Fig. S3A; Movie 8). This miniscule spectrosome globule at the anterior acted as a node for the addition of new spectrosome material, as it grew in size as the cycle proceeded. The dislodged spectrosomes, also known as ‘anchorless’ (López-Onieva et al., 2008), remained at the posterior until cytokinesis was completed, when they re-localised to the anterior of the GSC, fusing with the now conspicuous anterior spectrosome located next to the CpCs (Fig. S3B,C; Movie 9). Although the meaning of this novel spectrosome localisation in GSC behaviour is unknown to us, our results indicate that a significant proportion of GSCs possess posterior spectrosomes during a fraction of their cell cycle.

### Quantification of the GSC cell cycle using Fly-FUCCI and spectrosome morphology

**Fig. 3.**
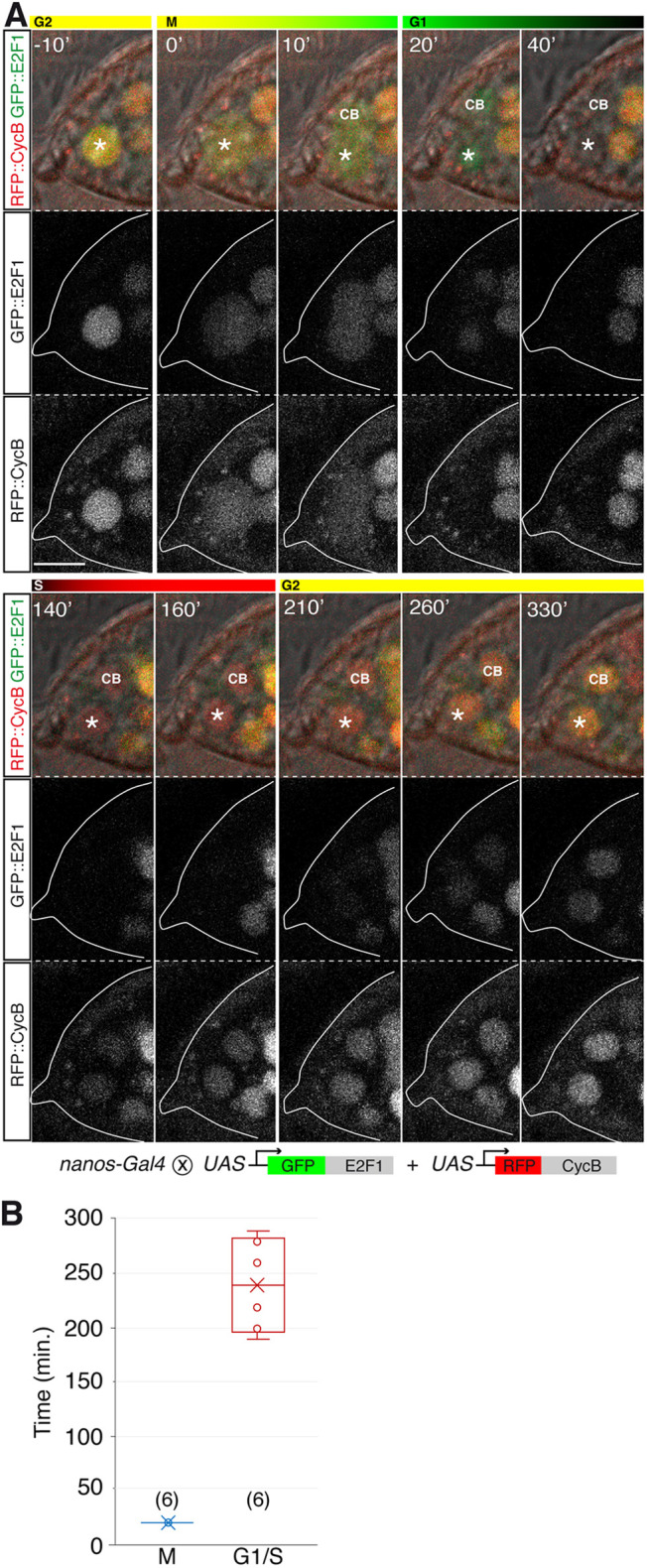
**Expression of Fly-FUCCI markers in live GSCs*.*** (A) *nanos>GFP:E2F1+mRFP1:NLS-CycB* germarium showing the cycling of GFP and RFP during a germline stem cell (GSC) division. Both GFP::E2F1 (green) and mRFP1::CycB (red) are present in G2 (t=−10′) and during the initial moments of M (t=0′). Only GFP::E2F1 remains in G1 (t=20′). During the rest of G1 (t=40′), GFP::E2F1 disappears and neither GFP nor mRFP1 are detected. In S phase, mRFP1::CycB reappears (t=140′). mRFP1::CycB and GFP::E2F1 are detected in G2 (t=210′). Asterisks indicate a GSC. CB, cystoblast. (B) Quantification of the duration of M and G1+S. The mean (cross) and median (line across box) values are shown. (*n*)=sample size. Scale bar: 10 μm. Related to Movie 10.

A number of studies have made use of cell cycle phase-specific markers to report that GSCs go through short G1, S and M, while G2 is the longest phase (Ables and Drummond-Barbosa, 2013; Hinnant et al., 2017; Hsu et al., 2008; Kao et al., 2015). To characterise the dynamics of cell cycle progression of female GSCs, we made use of the Fly-FUCCI tool, a strategy that uses GFP- and RFP-tagged degrons from E2F1 and Cyclin B proteins, respectively, to label G1 (only GFP::E2F1 is expressed), S (only RFP::CycB) and G2/M (both GFP::E2F1 and RFP::CycB) phases (Zielke et al., 2014) (Fig. 3A). We expressed these markers, which localise to the nucleus, in GSCs with the help of the *nanos-Gal4* driver (Van Doren et al., 1998) and filmed them for prolonged periods of time (up to 16 h). To avoid unnecessary bleaching of the GFP:E2F1 and RFP:CycB fusion proteins and to prevent photodamage of the samples, *z*-sections were collected at 1 μm intervals and *z*-stacks were recorded every 10 min. In spite of the long duration of these movies, we never managed to detect a GSC entering M phase twice, thus precluding us from determining the absolute duration of a GSC division cycle in our experimental conditions.

We observed live GSCs expressing only GFP:E2F1 (green cells, in G1), only RFP:CycB (red cells, in S) or both (yellow cells, in G2/M). GSCs undergoing mitosis could be distinguished because they transitioned from expressing nuclear GFP+RFP to releasing these markers to the cytoplasm (presumably at NEP; t=0′ in Fig. 3A; Movie 10), followed by the separation of the two daughter cells and the loss of the RFP signal (t=20′). In addition to the green, red and yellow cells, we also noticed GSCs that expressed neither GFP nor RFP and that were classified as ‘black’ GSCs. The study of six GSCs that underwent mitosis allowed the analysis of the FUCCI markers during complete M, G1 and S phases and of a portion of the G2 phase. These GSCs transitioned from GFP+RFP (G2/M) to only GFP (G1), to black, to only RFP (S) and to gradually GFP+RFP again (G2; Fig. 3A and Movie 10). Thus, our findings corroborated the predicted changes in the colour code of cycling cells and determined that ‘black’ GSCs were either in G1 or in S phases. On average, M phase in the Fly-FUCCI live movies lasted for 20 min while G1+S elapsed for 240 min±17.32 (Fig. 3B; *n*=6). Therefore, considering the estimated average of ∼15 h for a complete GSC division cycle in our culture conditions (Fig. 1D), M would take 3.3% of the total duration, G1+S 26.7% and G2 the remaining 70% (∼10.5 h).

**Fig. 4.**
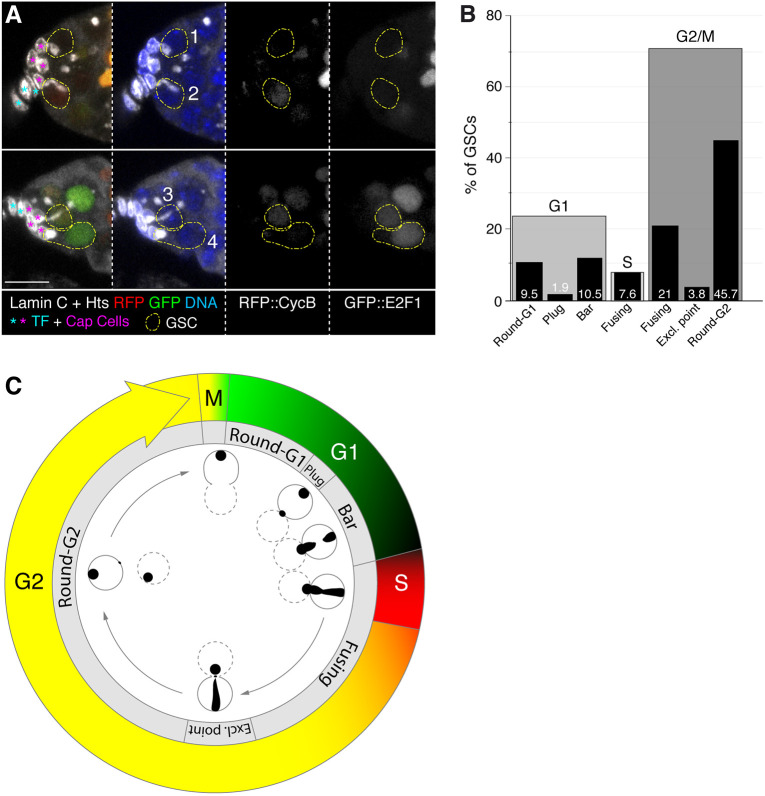
**Distribution of GSC spectrosomes and Fly-FUCCI markers in fixed niches.** (A) *nanos>GFP:E2F1+mRFP1:NLS-CycB* germaria stained with anti-Hts and anti-Lamin C (white) to label spectrosomes and fusomes, and cap cells, respectively. The RFP and GFP signals correspond to mRFP1::CycB and GFP::E2f1, respectively. (1) ‘Black’ GSC with a ‘round’ spectrosome, classified as in G1 phase; (2) RFP-positive germline stem cell (GSC) with a ‘fusing’ spectrosome (S phase); (3) RFP+GFP-positive GSC with an ‘exclamation point’ spectrosome (G2 phase); (4) GFP-positive GSC with a ‘round’ spectrosome (G1 phase). (B) Percentage of GSCs in G1, S or G2/M and the spectrosome morphologies associated with each of the cycle phases. Numbers in bars: percentage of GSCs displaying a given spectrosome morphology (*n*=105). (C) Representation of spectrosome and Fly-FUCCI marker dynamics throughout the cell cycle, including data on live and fixed GSCs. Dotted lines represent cystoblasts. Scale bar: 10 μm.

Next, we studied both the expression of the Fly-FUCCI markers and the different spectrosome morphologies in fixed GSCs. We examined carefully the Fly-FUCCI reporters in 105 GSCs from 37 different germaria co-stained with anti-Lamin C and anti-Hts antibodies to label CpC nuclear membranes, and the germline spectrosomes and fusomes, respectively. Of all GSCs examined, 70.5% expressed both GFP+RFP (G2/M; 74 cells), 10.5% only GFP (G1; 11 cells) and 3.7% only RFP (S; four cells). The remaining 15.2% (16 cells) did not show a detectable fluorescence and were classified, similarly to the *in vivo* Fly-FUCCI analysis, as ‘black’ GSCs (Fig. 4A). GFP cells (G1) showed ‘round’, ‘plug’ and ‘bar’ morphologies; RFP cells (S) were all ‘fusing’; and GFP+RFP GSCs (G2) contained ‘fusing’, ‘exclamation point’ and ‘round’ shapes. This is in agreement with previous reports (Ables and Drummond-Barbosa, 2013; Hinnant et al., 2017; Hsu et al., 2008; but see Kao et al., 2015). In order to ascribe the ‘black’ category to the different cycle phases, we studied the morphology of the 16 ‘black’ GSC spectrosomes: five displayed ‘round’ spectrosomes, two had ‘plugs’, five showed ‘bar’ figures and four contained ‘fusing’ spectrosomes. In accordance with our live Fly-FUCCI analysis, the spectrosome shapes present in ‘black’ GSCs also confirmed they were in G1/S. Furthermore, considering that none of the GFP-expressing cells contained ‘fusing’ spectrosomes and that all spectrosomes in RFP-expressing cells were ‘fusing’, we considered ‘black’ GSCs with ‘round’, ‘plug’ or ‘bar’ spectrosomes as cells in G1 phase, and the four GSCs with ‘fusing’ spectrosomes as belonging to S phase. Thus, of the entire batch of 105 fixed GSCs under study, 21.9% were in G1 (GFP or black with ‘round’, ‘plug’ and ‘bar’ spectrosomes), 7.6% in S phase (black or RFP with ‘fusing’ spectrosomes) and 70.5% in G2/M (GFP+RFP with ‘fusing’, ‘exclamation point’ or ‘round’ spectrosomes; Fig. 4B,C).

### The anterior GSC centrosome, which associates with the spectrosome in mitosis, separates immediately after division

**Fig. 5.**
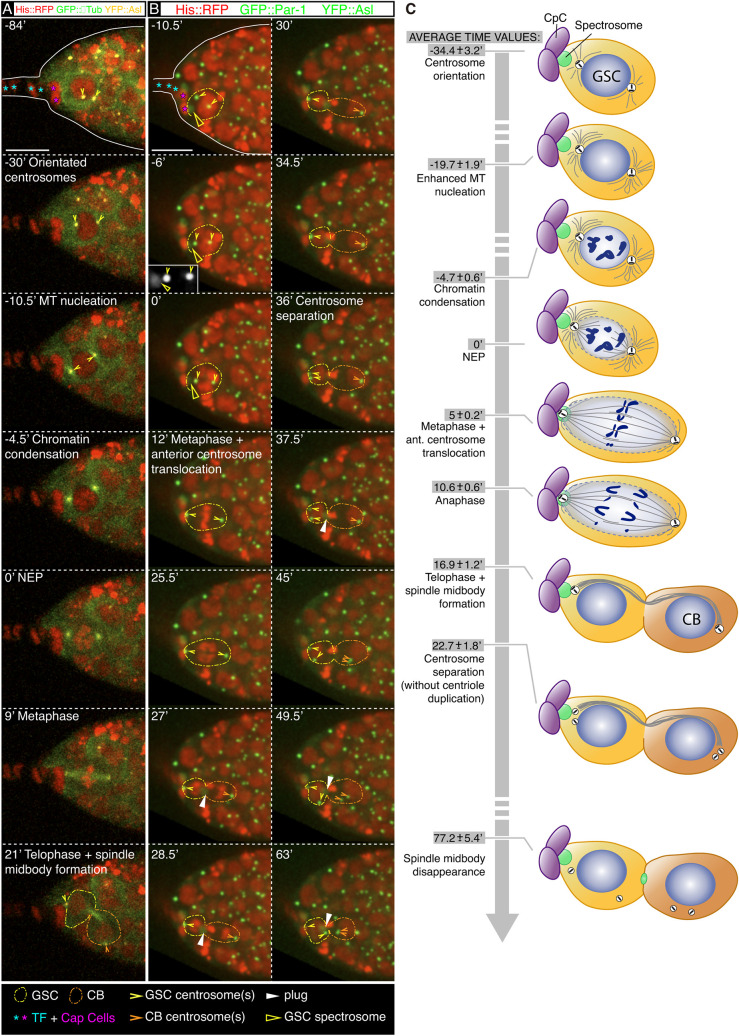
**The anterior GSC centrosome associates with the spectrosome in mitosis.** (A-C) Germline stem cell (GSC) centrosome separation takes place early in G1. (A) *YFP::asl, GFP::αtub, his::RFP* germarium to label centrosomes, microtubules and chromatin. (B) *YFP::asl, his::RFP, GFP::par-1* germarium showing the association of the anterior centrosome to the spectrosome (t=−6′), its subsequent translocation to the anterior cortex (t=12′) and GSC centrosome separation (t=36′; times refer to this particular example). Inset in t=−6′: higher magnification showing the signal from the spectrosome (GFP::Par1) and the GSC centrosomes (YFP::Asl). (C) Drawing of centrosomes (white), centrioles (black cylinders), microtubules (grey), cap cells (CpC, purple), spectrosome (green) and chromatin (dark blue) during GSC mitosis. CB, cystoblast; NEP, nuclear envelope permeation. The average starting time of each of the events (mean±s.e.m.) is shown. Sample sizes range from six to 14 (see the complete dataset in Fig. S4). Scale bar: 10 μm. Related to Fig. S4. Panel A related to Movie 11 and panel B to Movie 12.

To characterise GSC mitosis in greater detail, we imaged live germaria for 2 h with time points taken every 1.5 min. We expressed ubiquitously YFP::Asterless (YFP::Asl) to distinguish centrosomes, GFP::αTubulin (GFP::αTub) to label microtubules and Histone 2AV::mRFP (His::RFP) to mark chromatin. We identified 14 GSCs that underwent mitosis during the imaging and that allowed us to define three landmarks of GSC division, namely centrosome orientation, anterior centrosome translocation and centrosome separation after mitosis (Fig. 5). Before mitosis, centrosomes showed a dynamic behaviour (analysed in detail below) until they came to lie at opposite sides of the nucleus at −34.4 min±3.2 on average. Once centrosomes were orientated with respect to the nucleus, their microtubule-nucleating activity increased, as visualised by the enhanced GFP signal (−19.7 min±1.9). Next, chromatin condensation started (−4.7 min±0.6), followed by NEP (t=0′), mitotic spindle assembly and metaphase (5 min±0.2), anaphase (10.6 min±0.6) and telophase (16.9 min±1.2), which also marked the formation of the spindle MB, visualised as a condensed bundle of microtubules spanning the cytokinetic ring and connecting both daughter cells. The spindle MB disappeared nearly 1 h later on average (77.2 min±5.4; Fig. 5A,C; see Fig. S4 for individual data; Movie 11).

During GSC division, the mitotic spindle is orientated so that one of its poles faces the cap cells, a process that requires a functional spectrosome (Yamashita, 2018). To define in detail the interaction of the anterior centrosome with the mitotic round spectrosome, we filmed germaria expressing GFP::Par-1, YFP::Asl and His::RFP and observed that the anterior centrosome was adjacent to the spectrosome at the start of mitosis, as defined by the onset of chromatin condensation (*n*=4; Fig. 5B, t=−6′ in this particular example). Thus, the association of the anterior centrosome with the round spectrosome at the beginning of metaphase determines the final alignment of the centrosome pair with respect to the anterior-posterior axis of the germarium. This result is in line with previous reports that showed that one GSC centrosome is associated with the spectrosome in mitosis, but not in interphase (Deng and Lin, 1997; Salzmann et al., 2014; Stevens et al., 2007) but contradicts the findings of Lu et al. (2012), who claimed that the vast majority of GSCs had one centrosome associated to the CpC/GSC interface. Our movies also showed that, once the anterior centrosome came to lie next to the spectrosome, during metaphase it translocated to the presumptive cell cortex adjacent to the spectrosome (Fig. 5A, t=9′; 5B, t=12′). This appeared to be the anchoring point for the centrosome, as it remained there for the rest of mitosis.

The last of the three landmarks that characterised a GSC division was the separation of the GSC centrosome after mitosis. In all cases analysed, it took place few minutes after telophase, at 22.7 min±1.8 on average (Fig. 5B,C; Movie 12). Thus, the vast majority of the GSCs in a given niche should contain two centrosomes throughout most of their cell cycle. Interestingly, the CB centrosome separated almost at the same time as that of the GSC, providing further support for the proposed synchrony in the cell cycle of GSC/CB pairs at least until S phase (de Cuevas and Spradling, 1998).

### GSC centrosome separation in G1 occurs in the absence of centriole duplication

**Fig. 6.**
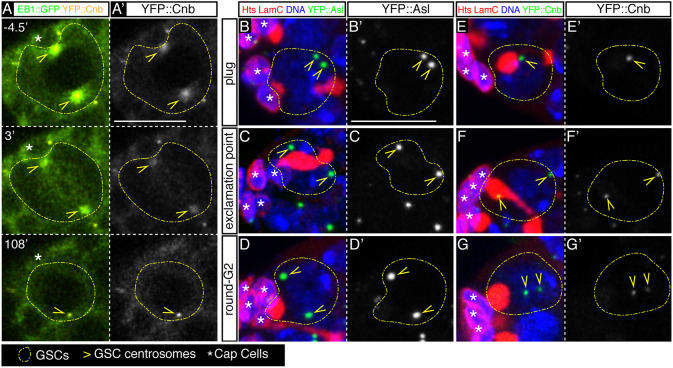
**GSC centrosomes separate before centriole duplication in S phase.** (A,A′) *EB1::GFP, YFP::cnb* germarium showing that the remaining germline stem cell (GSC) only contains one YFP::Cnb-positive centrosome 108 min after mitosis (t=0′ at nuclear envelope permeation). (B-G′) *YFP::asl* (B-D′) and *YFP::cnb* (E-G′) germaria stained with anti-Hts and anti-Lamin C (red) to label spectrosomes and fusomes, and cap cells, respectively, and with Hoechst to label nuclei (blue). The green signals in B-D label the localisation of both Asl-positive centrosomes. In panels E-G, the green signal indicates Cnb-positive centrosomes. Only cells with duplicated centrioles contain two Cnb-positive centrosomes. As all GSCs with ‘plug’ or ‘bar’ spectrosomes contain one Cnb-positive centrosome, whereas ‘exclamation point’ and ‘round-G2’ GSCs have two, centriole duplication occurs in S phase. Scale bars: 10 μm. Panel A related to Movie 13.

By the time an animal cell enters mitosis it harbours two active centrosomes. Thus, each sibling cell inherits one centrosome comprising the pericentriolar material plus an older ‘mother’ centriole and a younger ‘daughter’ centriole [the latter can be labelled using the Centrobin (Cnb) marker; Januschke et al., 2011; Zou et al., 2005]. Centrioles duplicate only once per cell cycle, normally in S phase and before centrosome separation, so that when centrosomes split before mitosis and migrate to opposite sides of the cell to form the spindle, each one of them carries a centriole pair. The finding that centrosome separation in the GSC occurs a few minutes after telophase indicated that centrosome splitting in these cells might take place before centriole duplication. We used YFP::Centrobin (YFP::Cnb) and EB1::GFP fusion proteins expressed under the *poly-Ubiquitin* promoter to label daughter centrioles and to track microtubule dynamics, respectively. Dividing GSCs showed two clear Cnb::YFP-positive dots associated with high levels of EB1::GFP signal, confirming that both mitotic centrosomes contained daughter centrioles. During metaphase, the YFP::Cnb signal faded away from the centrosomes and highlighted briefly the mitotic spindle before concentrating again on the centrosomes ∼10 min later. The single YFP::Cnb-positive dot in post-mitotic GSCs moved around the cytoplasm for the following 1.5 hours without duplicating (Fig. 6A; Movie 13). Because we have observed that post-mitotic GSC centrosomes separated on average ∼23 min after division, the above result strongly suggests that GSC centrosome separation occurs before centriole duplication. Finally, to define when during the cell cycle centrioles duplicated, we studied fixed GSCs labelled with the spectrosome marker Hts and either YFP::Asl or YFP::Cnb. We observed two YFP::Asl-positive dots in all of the GSCs analysed (*n*=40). In contrast, of the YFP::Cnb-expressing GSCs analysed (*n*=44), we identified nine GSCs that contained a single YFP::Cnb-positive centrosome (two with ‘round’ spectrosomes, two with ‘plug’ and five with ‘bar’), while the remaining 35 GSCs had two YFP::Cnb-positive dots (five ‘fusing’, three ‘exclamation point’ and 27 ‘round’; Fig. 6B-G). As all of the ‘plug’ or ‘bar’ GSCs (in G1) contained only one YFP::Cnb centrosome, and as all of the ‘fusing’ or ‘exclamation point’ (in S or G2) presented two, we concluded that centriole duplication in female GSCs takes place after centrosome separation, most likely early in S phase, as described for animal cells (Fu et al., 2015). Thus, GSCs with a ‘round’ spectrosome and a single Cnb-positive centrosome are in G1.

### The anterior and posterior centrosomes show distinct dynamics in pre-mitotic GSCs

**Fig. 7.**
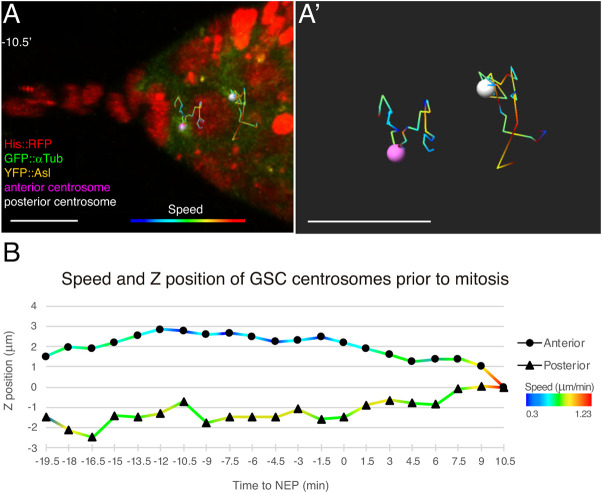
**Different dynamics of anterior and posterior GSC centrosomes before division.** (A) 3D rendition of a *GFP::asl, his::RFP, GFP::par-1* germarium in which the movement of both centrosomes has been tracked for 30 min. Tracks are colour-coded to reflect centrosome speeds (µm/min). (A′) Magnified view of the centrosome tracks. The anterior centrosome gradually restricts its movement until it associates with the anterior of the cell. The posterior centrosome moves longer distances and at a higher speed. (B) Quantification of the average speed and *z*-position of both centrosomes. The position of the centrosomes at the metaphase plate sets their *z*=0. The final time point corresponds to the formation of the metaphase plate (number of germline stem cells analysed=10). Scale bars: 10 μm. Related to Movie 14 and Fig. S5.

Whereas the orientation of the mitotic GSC centrosomes is undisputed (Deng and Lin, 1997; Salzmann et al., 2014), the position of GSC centrosomes during interphase is more controversial. Salzmann et al. (2014) and Stevens et al. (2007) reported that interphase centrosomes were not orientated with respect to the niche, whereas Lu et al. (2012) proposed that female GSCs behaved like their male counterparts, which have one centrosome always positioned next to the niche/GSC interface (Yamashita et al., 2003). Our recording of centrosome movements during the GSC cell cycle suggested that the two interphase centrosomes were randomly positioned within the cell and that it was only before mitosis that their positions became more constrained, particularly that of the anterior centrosome, which restricted its movement to the anterior hemisphere of the cell (Fig. 7A; Movie 11). Once both centrosomes became orientated on opposite sides of the nucleus, they limited their movement but still showed some variations in their positions (Movie 14). We analysed in detail the dynamics of centrosome positioning and quantified the position along the *z*-axis and the speed of displacement of both the anterior and posterior centrosomes of 10 GSCs from −20 min to +10 min, when the metaphase plate was clearly formed (NEP, t=0′). The final position of each of the centrosomes at the metaphase plate was considered the *z*=0 point for both of them. The initial *z* position of the anterior centrosome was given a positive value and the subsequent *z* positions of the anterior and posterior centrosomes were calculated accordingly. Our results showed that, on average, both centrosomes moved within a 3 µm range and that the anterior centrosome gradually restricted its movements to the GSC area facing the CpC rosette. In contrast, the posterior centrosome moved at higher speeds until it reached its final position (Fig. 7B; Fig. S5; Movie 14). We concluded that GSC centrosomes are quite dynamic, even once they are placed on both sides of the nucleus before division, and that the anterior centrosome only fixes its position upon its association with the anterior spectrosome.

### Tumour GSCs divide symmetrically

**Fig. 8.**
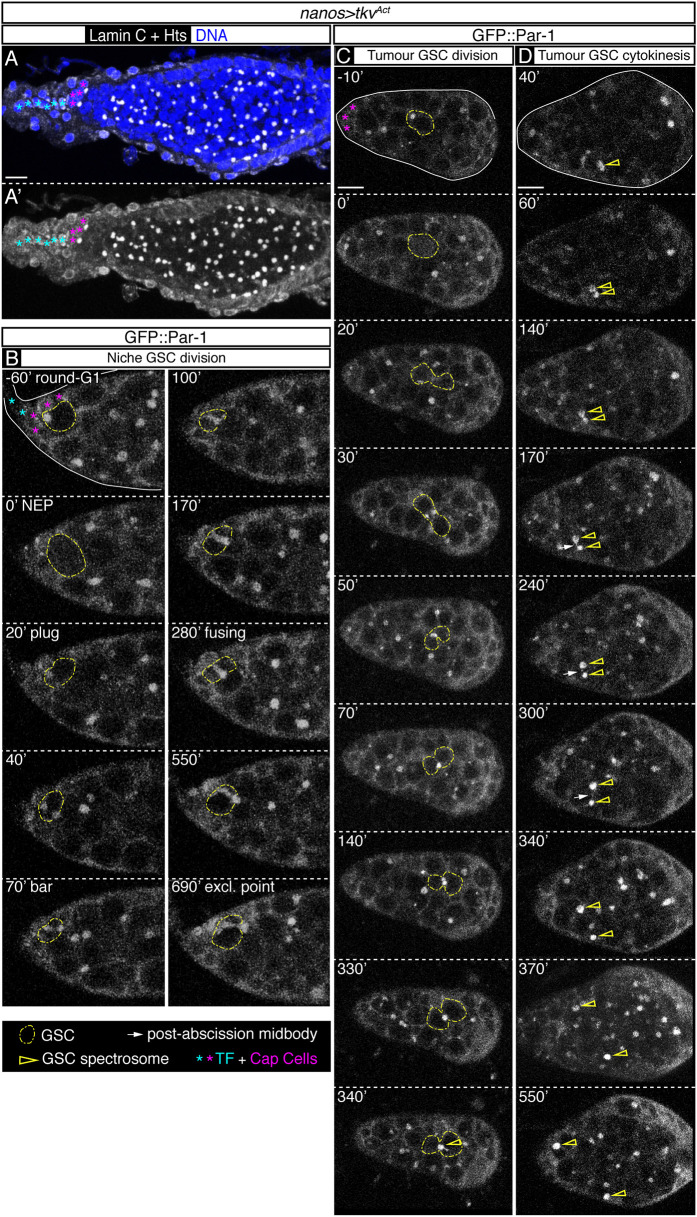
**The spectrosome in GSC-like tumours divides symmetrically.** (A) *nanos>tkv^Act^* germarium stained with anti-Hts and anti-Lamin C (white) to label spectrosomes and fusomes, and cap cells, respectively, and Hoechst to mark nuclei (blue). The germarium is filled with germline stem cell (GSC)-like cells containing only ‘round’ spectrosomes, but it lacks branched fusomes, indicative of differentiating cysts. (B,C) *GFP::par-1*, *nanos>tkv^Act^* germaria showing the typical asymmetric division of a GSC inside the niche from ‘round-G1’ until the ‘exclamation point’ phase (t=0′, NEP) (B) and the division of a GSC-like tumour cell outside the niche (C). The spectrosome is initially opposite the place of cytokinesis. During mitosis (t=0′, NEP), the spectrosome moves to the cytokinetic ring (t=20′) and starts accumulating new material (see t=340′). (D) Final steps in the cytokinesis of two daughter cells. Upon cytokinesis (t=60′), the post-abscission midbody is generated (shown at t=170′). The sister cells rapidly drift apart several cell diameters (t=240-550′). NEP, nuclear envelope permeation. Scale bars: 10 μm. Panels B and C related to Movies 15 and 16.

Our work and that of many others has determined that spectrosomes divide asymmetrically between sibling GSCs and CBs. Whether this is an intrinsic property of the GSCs themselves or it is a behaviour dictated by the surrounding niche remains an open question. Thus, we embarked upon the analysis of GSC tumours in which ectopic GSC-like cells can be found several cell diameters away from the niche, as defined by the anterior position of the TF and CpCs (Fig. 8A). To generate these masses of ectopic GSCs, we expressed an activated form of the Dpp receptor *thickveins* in the germline using the *nanos-Gal4* line (*nanos>tkv^Act^*) (Casanueva and Ferguson, 2004). To be able to follow live the division of the ectopic GSCs we used the GFP::Par-1 marker.

We performed long duration movies (up to 16 h) of *GFP::par-1+nanos>tkv^Act^* ovaries and analysed the occurrence of GSC divisions both inside the niche and at a distance from it. First, we confirmed that GSCs hosted within the niche showed stereotypic divisions indistinguishable from the ones found in control niches (Fig. 8B; Movie 15). We then focused on the behaviour of the GSCs found in the large tumours, which showed a high degree of cell movements, as it was commonplace to observe GSCs continuously changing positions, even without going through mitosis. We managed to identify five GSCs that underwent mitosis and seven germline pairs that finished cytokinesis in the movies. We concluded that the spectrosome was located at one end of the cell, opposite to the future cytokinesis site. After the ensuing mitosis, the spectrosome was re-positioned to the cytokinetic ring, where it remained for several hours, time during which new spectrosome material was added to the growing organelle in both daughter cells (Fig. 8C; Movie 16). Finally, after 4-6 h the spectrosome was split between both cells, each daughter inheriting a similarly-sized spectrosome. In contrast to the GSCs in contact with the CpCs, which inherit the post-abscission MB, the MB in GSC tumours did not appear to associate to any of the daughter cells and was eventually lost in the movies (Fig. 8D; Movie 16). From these observations we concluded that the tumorous GSC-like cells present in *nanos>tkv^Act^* germaria that are not in the niche divide their spectrosome in a symmetrical fashion.

## DISCUSSION

### Polarised vesicle transport in the GSCs

Here, we show that the asymmetric self-renewing division of live GSCs is reflected in the behaviour of the spectrosome and the inheritance of the post-abscission MB, as the GSCs remaining in the niche retain most of the spectrosome material and inherit the MB remnant. The continuous growth of the GSC and CB spectrosomes suggests that these cells are actively synthesising spectrosome material and points to a polarised intracellular trafficking responsible for the transport towards the enlarging spectrosomes. In fact, the membrane vesicles observed in spectrosomes and fusomes resemble those of the endoplasmic reticulum or the Golgi apparatus (Lighthouse et al., 2008; Röper, 2007; Snapp et al., 2004). In the case of the anterior spectrosome adjacent to the CpC rosette, it is known that the active form of the small GTPase Rac accumulates at the niche-GSC interface and brings about the microtubule binding protein Apc2 to orientate the mitotic spindle (Lu et al., 2012). Although we have not observed a defined organisation of the interphase microtubules that may account for this polarisation within the GSC, it has been reported that the niche-GSC interface possesses a higher concentration of microtubules and that actin and microtubule-interacting proteins such as Par-1 accumulate at the spectrosome. In fact, a polarised trafficking of Rab11-positive recycling endosomes has been proposed to account for the apical placement of the spectrosome and to keep a proper DE-cadherin-based GSC/CpC adhesion (Bogard et al., 2007). Whether the GSC uses this intrinsic polarity to organise the spectrosome transport machinery remains to be tested but the CpC/GSC interface most likely plays an essential role, as even dislodged spectrosomes that undergo abscission at the posterior are translocated to the anterior to fuse with the ‘scar’ of spectrosome material found adjacent to the CpC rosette. In this regard, it is convenient to emphasise the importance of the CpC/GSC interaction for the establishment of the GSC anterior-posterior polarity, as GSC-like cells found several cell diameters away from the niche divide symmetrically, at least as shown by their spectrosome behaviour. This would indicate that, in the case of the female GSCs, the asymmetric outcome of their division relies largely on the microenvironment.

### A role for Par-1 in the regulation of GSC division?

The localisation of the Par-1 kinase in the female GSC may indicate a possible function in the regulation of the cell cycle. Par-1, like the Hts protein, is a major component of spectrosomes and fusomes, as it decorates the differently shaped spectrosomes characteristic of G1, S and G2 phases and the fusomes of germline cysts. It also labels the new material that fuses with the growing spectrosomes present in GSCs and CBs. During mitosis, however, Par-1 loses its spectrosome association and moves to the cytoplasm for a brief period before regaining its spectrosome localisation, a feature shared also by differentiating fusomes. Interestingly, male GSCs – which also contain Par-1 in interphase spectrosomes and lose it during mitosis (Yuan et al., 2012) – possess a centrosome orientation checkpoint (COC) that ensures their asymmetric division. The COC arrests the GSC cell cycle in G2 if centrosomes are not orientated properly (Venkei and Yamashita, 2015; Yamashita, 2018; Yuan et al., 2012). Par-1 is an important component of the COC, where it acts to ensure that CycA localises to the spectrosome during G2, thus preventing precocious entry into mitosis when centrosomes are not properly aligned. Upon the onset of prophase, CycA is released from the spectrosome and is quickly degraded by metaphase (Yuan et al., 2012). In the ovary, CycA is also associated with the spectrosome/fusome in G2/M (Lilly et al., 2000), opening the possibility that Par-1 controls the GSC cell cycle via CycA localisation to the spectrosome in G2 and releasing it during mitosis. Alternatively, as the Par-1 kinase is released from the spectrosome in GSCs and CBs (and the fusome in older cysts) precisely during the rearrangement of the microtubule cytoskeleton in mitosis, and as Par-1 is involved in the regulation of microtubule organisation (Cox et al., 2001; Huynh et al., 2001; Shulman et al., 2000), Par-1 could be required for the correct setting of the microtubule network in dividing germline cells.

### The centrosome cycle in female GSCs

The canonical view of the centrosome cycle in eukaryotic cells states that centriole duplication takes place during S phase and that centrosomes separate before mitosis. Thus, cells during G1 and S phases contain only one centrosome. The study of centrosome behaviour in a variety of stem cell types has yielded striking differences. For example, centrosomes in *Drosophila* neuroblasts (NBs), stem cells of the fly central nervous system, separate during mitotic exit and before centriole duplication. Both the mother and daughter centrosomes display different microtubule-nucleating activities, with the daughter centrosome being more active. Moreover, the remaining NB retains the centrosome containing the daughter centriole, whereas the sibling ganglion mother cell inherits the mother centriole (Conduit and Raff, 2010; Januschke et al., 2011; Rebollo et al., 2007; Rusan and Peifer, 2007). In male GSCs, centrosome separation occurs in G2, after centriole duplication in S phase (Yamashita et al., 2007), and centrosomes are orientated so that the mother centrosome is always positioned next to the niche cells. Thus, upon asymmetric division the remaining GSC inherits the mother centrosome. Our results demonstrate that female GSCs behave more like NB, as they separate their centrosomes very early in G1, before centriole duplication. We have not determined which centrosome is retained by the GSC, but it had been postulated that it is the daughter one (Salzmann et al., 2014). However, we believe this should be revisited. The original observation was based on the fact that in GSCs with a ‘round’ spectrosome and with orientated centrosomes in which one was Cnb-positive and the other Cnb-negative, the former associated with the spectrosome in the vast majority of cases. The authors assumed that these GSCs contained immature centrosomes with only one centriole each and that they were in G2, and concluded that the daughter centrosome was ‘preferentially inherited by the female GSC’. Our results show that the only GSCs with one Cnb-positive centrosome and with a ‘round’ spectrosome are in early G1. Thus, the anterior localisation of the daughter centrosome (containing the Cnb-positive centriole) does not necessarily indicate that it will remain in the GSC.

### The cell cycle in female GSCs

Our analyses of live and fixed samples rendered consistent average durations for the cell cycle phases as determined by the spectrosome morphologies, the behaviour of chromatin markers and the Fly-FUCCI colour codes. Thus, M lasted for 2.4% of the cycle in live movies; G1 (‘round-G1’, ‘plug’ and ‘bar’ spectrosome shapes) was 24.7% in live samples and 21.9% in fixed tissues; S lasted for 4.7% in live samples and 7.6% in fixed germaria (these values were calculated considering that, according to the fixed Fly-FUCCI data, nearly 27% of GSCs with ‘fusing’ morphologies were in S phase, and the remaining 73% in G2); and G2 68.5% in live samples and 70.5% in fixed ones. Considering that in our experimental conditions, a GSC divides on average every ∼15.5 h, M lasts for 22 min, G1 for ∼3.45 h, S for ∼43 min and G2 for ∼10.5 h. However, the variability in the proliferation time of individual GSCs may be considerable, given the dispersion – with the exception of the M phase – in the duration of the different spectrosome morphologies observed in live samples. The relatively short G1 and long G2 phases characteristic of female GSCs are shared with other stem cell types but, while the cell cycle control of functional GSCs is important for their stemness, the molecular details of how this is achieved are not known (Hinnant et al., 2020). Interestingly, it has been postulated that stem cells possess a short G1 to retain a naïve, pluripotent state and that the ratio of S phase to the gap phases changes with the differentiation status of the cell. In fact, murine embryonic stem cells have a relatively short G1 phase and it is thought that they are vulnerable to differentiation cues in G1 (Pauklin and Vallier, 2013). Whether the *Drosophila* female GSCs implement a similar strategy and possess a short G1 to prevent unwanted differentiation awaits further investigations.

## MATERIALS AND METHODS

### Fly stocks

Flies were grown at 25°C on standard medium. The lines used include: *GFP::par-1: w;; pUbi-GFP::par-1* (this work); *w; nanos-G4* [Bloomington *Drosophila* Stock Center (BDSC), 4442]; *w; UASp-GFP:E2F1, UASp-mRFP1:NLS-CycB/CyO, wg-lacZ* (BDSC, 55110); *YFP::asl: w, pUbi-YFP::asl* (Rebollo et al., 2007); *GFP::αtub: w, pUbi- GFP::αtubulin 84B* (Rebollo et al., 2004); *his::RFP: w;; His2AV::mRFP1* (BDSC, 25377); *YFP::cnb: w; pUbi-YFP::cnb/CyO* (Januschke et al., 2011); *EB1::GFP: w;; pUbi-EB1::GFP/TM6B* (Shimada et al., 2006); *w;; UASp-Tkv^Act^/TM3* (Casanueva and Ferguson, 2004).

### Generation of pUbi-GFP::par-1 flies

To create *P{poly Ubiquitin-mGFP6::par-1}*, flies were transformed with a pWhiteRabbit vector (a gift from Prof. Nick Brown, University of Cambridge, UK) containing a 3.5 kb fragment of the *mGFP6::par-1* construct (Huynh et al., 2001) flanked by *Kpn*I and *Not*I sites and downstream of the *poly-Ubiquitin* promoter. The resulting construct was verified by restriction digests and sequencing. Transgenic lines were generated by standard procedures (Rubin and Spradling, 1982).

### *Ex vivo* culturing conditions for germaria

Before dissection, 1-2-day-old females were yeasted for 2 days. Cultures were prepared differently depending on the length of the movies. For short movies (up to 2 h long) we followed the protocol by Valencia-Expósito et al. (2016) with slight modifications. Briefly, ovaries were dissected in Schneider's medium (Biowest, L0207-500) supplemented with 15% (v/v) foetal bovine serum (Gibco, 10500-064; S-FBS), 0.6% (v/v) streptomycin/penicillin antibiotic mix (Invitrogen, 15140-122) and 0.20 mg/ml insulin (Sigma-Aldrich, 15500). Individual ovarioles without the muscle sheath were transferred in a small volume (1-2 μl) of supplemented Schneider's medium to a 35-mm poly-D-lysine-coated plate (MatTek, P35GC-1.5-10-C). Ovarioles were then mounted in 100 μl of 2% (w/v) low-melting point agarose poured in a plastic ring sealed to the bottom of the MatTek plate with vacuum grease (see Fig. S1A). Once the agarose solidified, the plate was filled with supplemented Schneider's medium until the ring and the agarose inside were fully covered.

In the case of 10- to 16 h-long movies, ovaries were dissected in Ringer's medium [128 mM NaCl, 2 mM KCl, 1.8 mM CaCl_2_, 4 mM MgCl_2_, 35.5 mM sucrose, 5 mM HEPES (pH 6.9)]. Isolated ovarioles without the muscle sheath were transferred in a small volume of Ringer's medium to a MatTek plate in which the 35-mm poly-D-lysine-coated plate was additionally covered with Cell-Tak (Corning 354240). Before ovary dissection, a 3-μl Cell-Tak drop was placed in the centre of the MatTek coverslip bottom, without manual spreading, and an equal volume of 0.1 M NaCOH_3_ was carefully mixed into the Cell-Tak drop and allowed to evaporate at room temperature. Transferred ovarioles were gently but quickly pressed against the Cell-Tak cover using a dissection needle. The plate was then half-filled with supplemented Schneider's medium (Fig. S1A). We chose 10-min time intervals in order to minimise bleaching and photodamage of the samples while at the same time trying to achieve an informative time-resolution. We also tested 5-min intervals, but the bleaching of the signal after a few hours was not worth the increase in time resolution of the movies.

### Immunohistochemistry

Adult flies were yeasted for 2 days before dissection in PBT (phosphate buffered saline+0.1% Tween 20). Ovary stainings were performed at room temperature. Ovaries were fixed in 4% paraformaldehyde in PBT for 20 min, washed in PBT for 20 min and blocked in 10% bovine serum albumin (BSA) in PBT for 1 h. Incubation with primary antibodies was performed overnight at the following concentrations: mouse anti-Hts [1B1, Developmental Studies Hybridoma Bank (DSHB), 1:100] and mouse anti-LaminC (LC28.26, DSHB, 1:30). Secondary antibodies Cy3 and Cy5 (Cy3 RRID: AB_2340813; Cy5 RRID: AB_2340820; Jackson Laboratories; final concentrations of 1:100) and conjugated anti-GFP-488 nanobody (gba488, Chromotek, 1:200) were incubated for 4 h. To stain DNA, ovaries were incubated for 10 min with Hoechst (Sigma-Aldrich, 5 mg/ml; 1:1000 in PBT).

### Imaging of fixed samples

Images were acquired using a Leica SP5 confocal microscope, analysed using Imaris and ImageJ, and processed with Adobe Photoshop and Adobe Illustrator. *Z*-stacks of fixed samples were taken at 0.7 μm intervals using a 63×/1.4 NA oil immersion objective.

### Imaging of live samples

With the exception of Figs 5B, 6A and Movies 12 and 13, which were captured using a PerkinElmer UltraVIEW VoX spinning-disk microscope (only two colours were captured, YFP and GFP shown in green, and RFP, shown in red), images were acquired using a Leica SP5 confocal microscope, analysed using Imaris and Fiji (Schindelin et al., 2012), and processed with Adobe Photoshop and Adobe Illustrator. *Z*-stacks of live samples were taken at 1.2 μm intervals and time points recorded every 1.5 or 10 min using a 40×/1.3 NA oil immersion objective.

### Data analysis

The different GSC spectrosome shapes were identified according to previous descriptions (de Cuevas and Spradling, 1998; LaFever et al., 2010). We used the following landmarks for each of the five spectrosome morphologies: ‘round-G1’ corresponds to a single sphere abutting the anterior CpC rosette; ‘plug’ refers to the anterior sphere plus a disk of spectrosome material filling the GSC-CB intercellular bridge; ‘bar’ commences when the intercellular disk grows and becomes ovoid in shape; ‘fusing’ results from the fusion between the anterior sphere and the middle ovoid disk as it stretches from the anterior margin of the GSC into the future CB; ‘exclamation point’ begins the moment at which abscission takes place, strangling the spectrosome connecting the GSC and the CB, and lasts until the elongated spectrosome material recoils to become spherical again, which corresponds to ‘round-G2’.

To quantify the fluorescent signal of GFP::Par1 in dividing GSCs, we defined regions of interest (ROIs) in spectrosomes and in nuclei during five time points before and five time points after, NEP (−50′ to 50′). For each ROI, we measured the mean intensity value of the GFP channel. The t=−50′ values were considered 100% and used as reference for the rest of the collected values.

To quantify the endogenous fluorescent signal of Fly-FUCCI markers, we selected the *z*-section containing the largest nuclear diameter for each of the GSCs. The selected nuclei were manually delineated and mean intensity values were measured for the GFP (E2F1) and RFP (CycB) channels. Background signal was subsequently subtracted and an intensity value threshold was established to classify GSCs as positive or negative for each marker.

### Statistical analysis

Experiments were performed with at least three biological replicates. Samples were collected from at least five different adult females grown in equivalent environmental conditions. Average values shown correspond to the arithmetic mean and the standard error of the mean (s.e.m.) of the different experimental settings. Sample sizes correspond to the number of GSCs analysed or to the number of events quantified. In all of the box and whisker plots, the box corresponds to the first and third quartiles. The upper whisker extends from the third quartile to the highest value. The lower whisker extends from the first quartile to the lowest value.

### Experimental genotypes

Fig. 1: (B,D,E) *w;; pUbi-GFP::par-1*.

Fig. 2: (A,B,C) *w;; pUbi-GFP:: par-1*.

Fig. 3: (A,B) *nanos>GFP:E2F1+mRFP1:NLS-CycB: w; nanos-Gal4*/*UASp-GFP:E2F1, UASp-mRFP1:NLS-CycB*.

Fig. 4: (A,B) *nanos>GFP:E2F1+mRFP1:NLS-CycB: w; nanos-Gal4*/*UASp-GFP:E2F1, UASp-mRFP1:NLS-CycB*.

Fig. 5: (A) *w, pUbi-YFP::asl, pUbi-GFP::αtubulin 84B; His2AV::mRFP1*; (B) *w, pUbi-YFP::asl; His2AV::mRFP1; pUbi-GFP:: par-1*.

Fig. 6: (A) *w; pUbi-YFP::cnb/CyO*; *pUbi-EB1::GFP/TM6B*; (B,D,F) *w, pUbi-YFP::asl*; (C,E,G) *w; pUbi-YFP::cnb/CyO*.

Fig. 7: (A) *w, pUbi-YFP::asl, pUbi-GFP::αtubulin 84B; His2AV::mRFP1*.

Fig. 8: (A-D) *GFP:: par-1, nanos>tkv^Act^: w; nanos-Gal4/+; UASp-Tkv^Act^/pUbi-GFP:: par-1*.

Fig. S1: (B,D) *y w*; (C) *w;; pUbi-GFP:: par-1*.

Fig. S2: (A) *w;; pUbi-GFP:: par-1*; (B) *y w*.

Fig. S3:*w;; pUbi-GFP:: par-1*.

Movies 1-9: *w;; pUbi-GFP:: par-1*.

Movie 10:*w; nanos-Gal4*/*UASp-GFP:E2F1, UASp-mRFP1:NLS-CycB*.

Movies 11 and 14: *w, pUbi-YFP::asl, pUbi-GFP::αtubulin 84B; His2AV::mRFP1*.

Movie 12:*w, pUbi-YFP::asl; His2AV::mRFP1; pUbi-GFP:: par-1*.

Movie 13:*w; pUbi-YFP::cnb/CyO*; *pUbi-EB1::GFP/TM6B*.

Movies 15 and 16: *w; nanos-Gal4/+; UASp-Tkv^Act^/pUbi-GFP::par-1*.

## Supplementary Material

Supplementary information

Reviewer comments
