# Supplementary material for: Live imaging of the Drosophila ovarian niche shows spectrosome and centrosome dynamics during asymmetric germline stem cell division
Source: Development. 2021 Sep 17;148(18):dev199716. doi: 10.1242/dev.199716 (PMC8489027; doi:10.1242/dev.199716)
Supplement: Supplementary information [file develop-148-199716-s1.pdf]

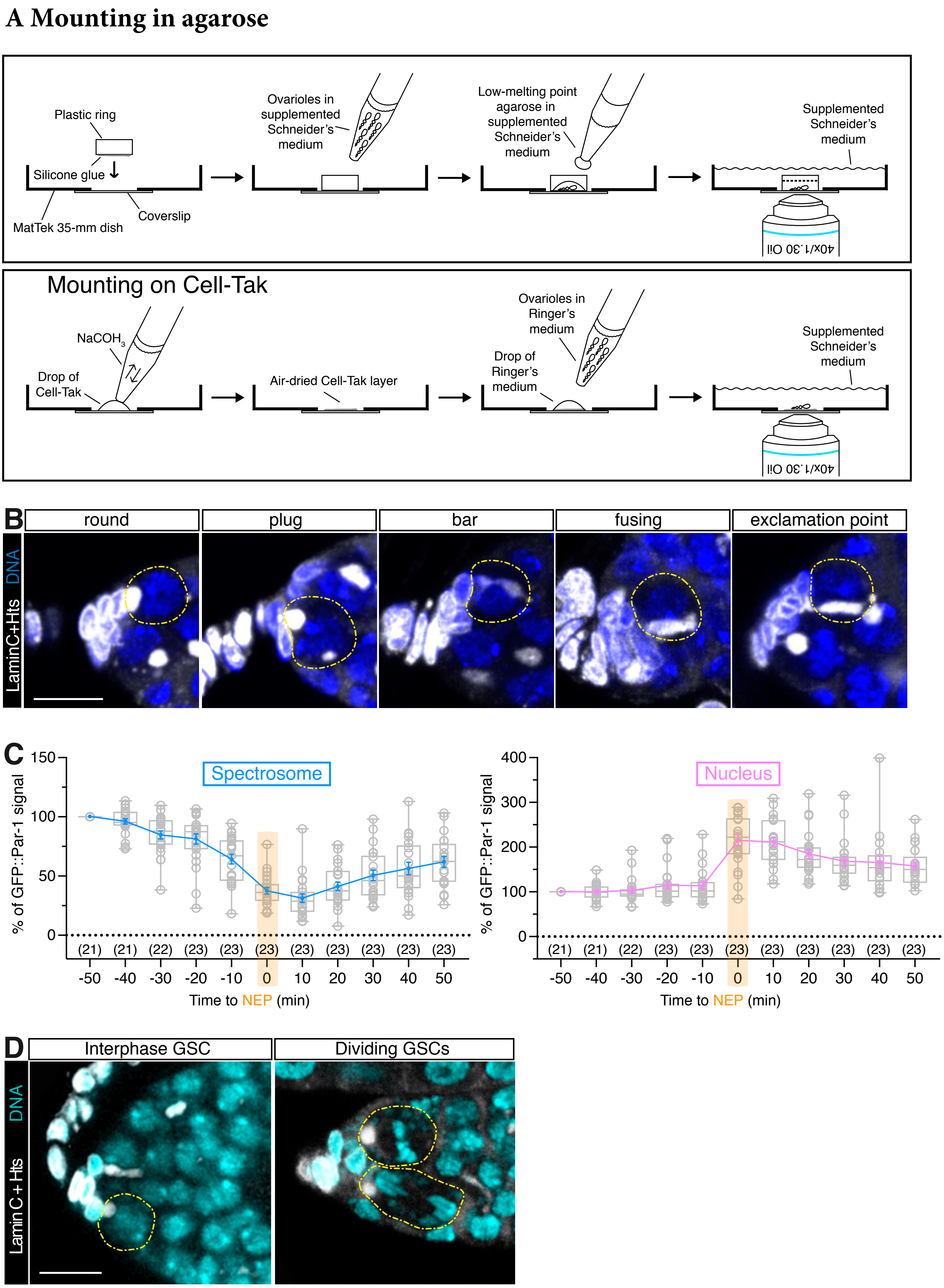

Development • Supplementary information

Fig. S1. Mounting methods for *ex vivo* imaging. Spectrosome morphologies observed in mitotic and interphase GSCs. The round spectrosome is maintained during GSC mitosis. (A) Schematic representations of the basic steps for mounting ovarioles in agarose (we used this method for short movies, up to 2 hours long) or in Cell-Tak (this method was utilised with 10-16-hour-long movies; see Materials & Methods for details). **(B)** Control germaria stained with anti-Hts and anti-Lamin C to label spectrosomes and cap cells, respectively (white), and Hoechst to mark nuclei (blue). The panels show the five spectrosome morphologies identified in live germaria. **(C)** Quantification of the GFP signal in spectrosomes and nuclei of 23 GSCs, from t= -50’ to t= 50’ (NEP, t= 0’). The individual values of each of the measurements taken are represented in the box and whisker graphs. t= -50 measurements were given a 100% value, which were then used to normalise the rest of the data points. Coloured lines represent the MEAN; error bars correspond to the SEM. Digits in parenthesis refer to the number of spectrosomes or nuclei analysed in each of the 11 time points. **(D)** Control germaria stained with anti-Hts and anti-Lamin C to label spectrosomes and cap cells, respectively (white), and Hoechst to mark nuclei (cyan). Yellow, dotted lines demarcate GSCs with round spectrosomes. Both the interphase GSC (left panel) and the dividing GSCs (right panel) display a clear round spectrosome, showing that this organelle is not disassembled in mitosis. Scale bars: 10 µm. Related to Figures 1 and 2.

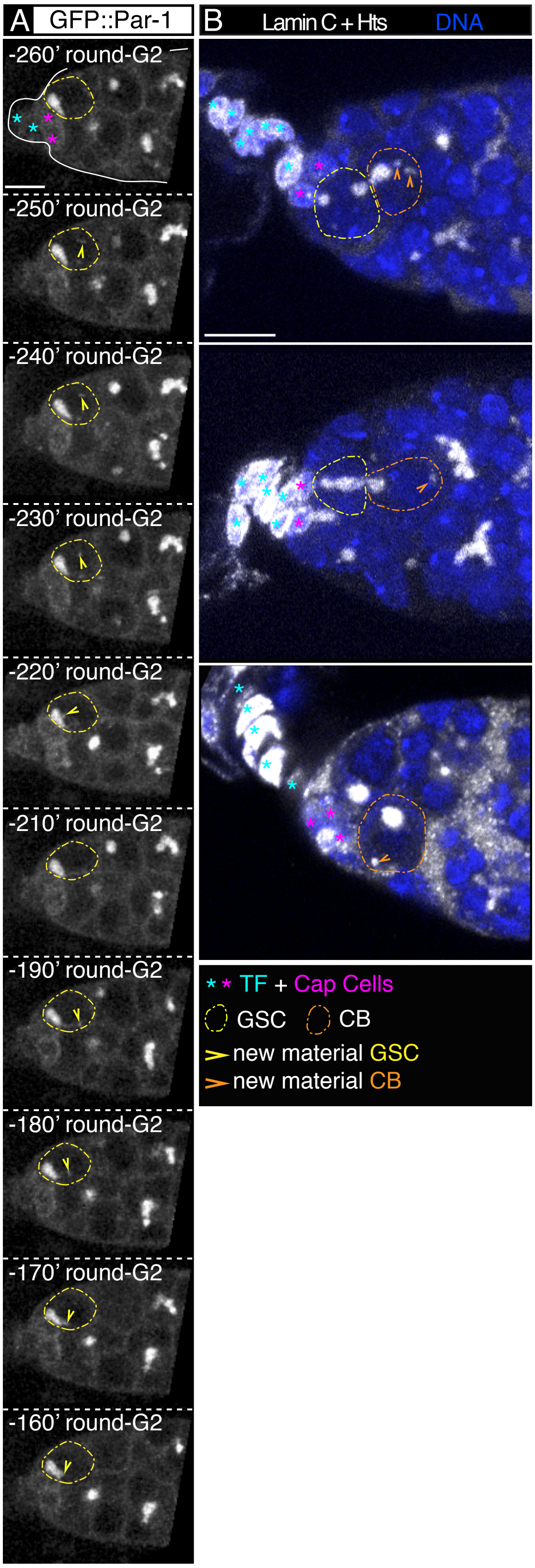

Development • Supplementary information

Fig. S2. Incorporation of new material into "round-G2" spectrosomes. Fixed CBs also contain GFP::Par-1 positive vesicles. (A) Time lapse stills of a *GFP::par-1* germarium showing the addition of new material to a "round-G2" spectrosome. **(B)** Control germaria stained with anti-Hts and anti-Lamin C to label spectrosomes and fusomes + cap cells, respectively (white), and Hoechst to mark nuclei (blue). Fixed germaria also display GSCs and CBs containing Hts-positive vesicles that resemble those observed in live cells. Presumably, these vesicles are transported towards, and fused with, the spectrosomes. Scale bars: 10 µm. Related to Figure 2 and Movie S5.

Development • Supplementary information

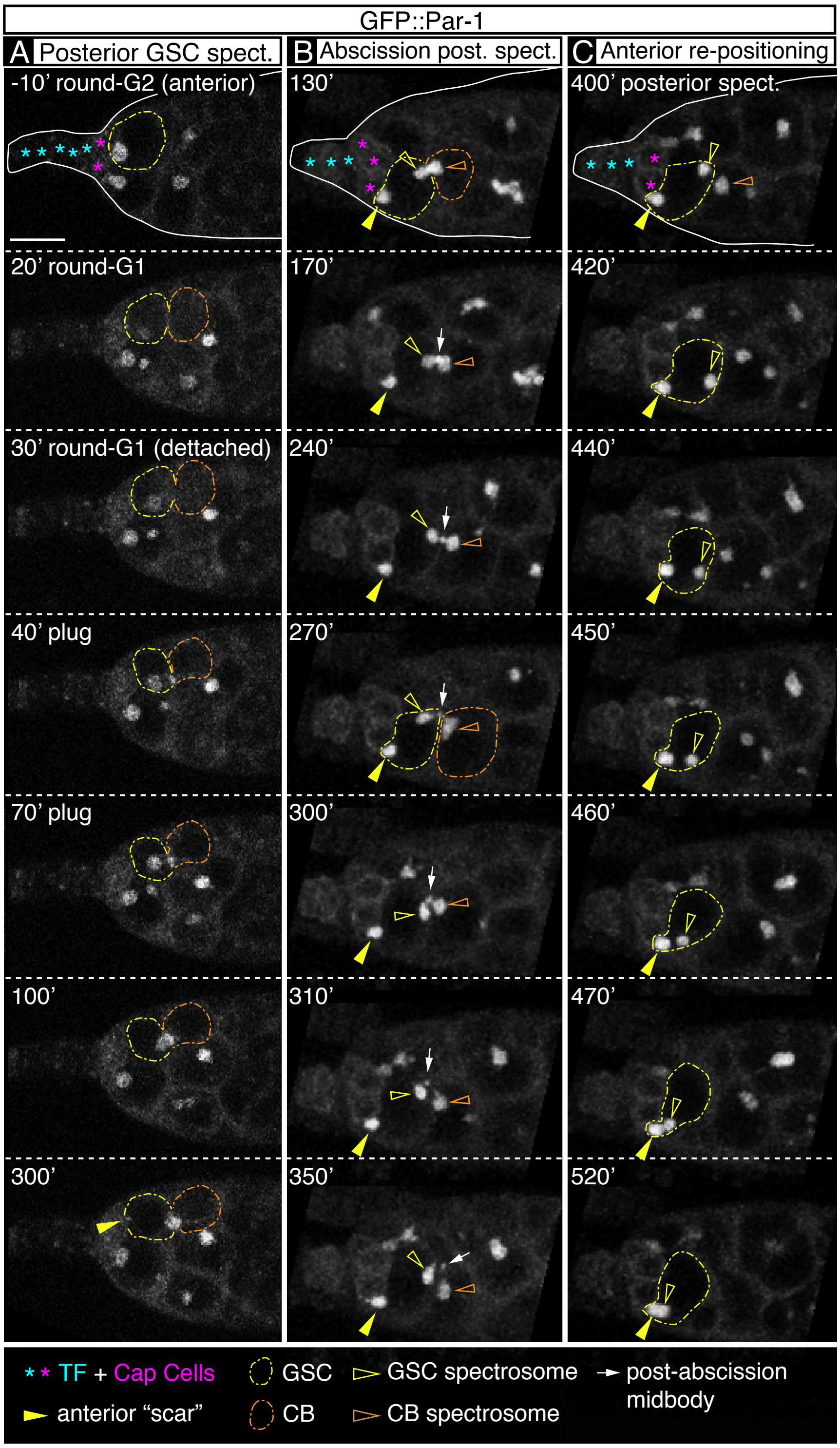

Development • Supplementary information

Figure S3: Non-canonical GSC spectrosome behaviour: posterior spectrosomes. (A) Time lapse stills of a *GFP::par-1* germarium showing a dividing GSC in which the spectrosome does not remain at the GSC/CpC interface. Rather, it detaches and moves to the posterior pole of the cell. Note the anterior "scar" of spectrosome material. It seems to act as a seed for the accumulation of new material during the cycle. **(B, C)** Time lapse stills of a *GFP::par-1* germarium showing (B) a GSC with an anterior scar and a posterior spectrosome undergoing cytokinesis (t= 130-350'). (C) The posterior spectrosome is then repositioned to the anterior margin of the GSC, where it fuses with the anteriorly placed spectrosome (t= 400-520'). Scale bar: 10 µm. Related to Figure 2 and Movies S8 and S9.

Development • Supplementary information

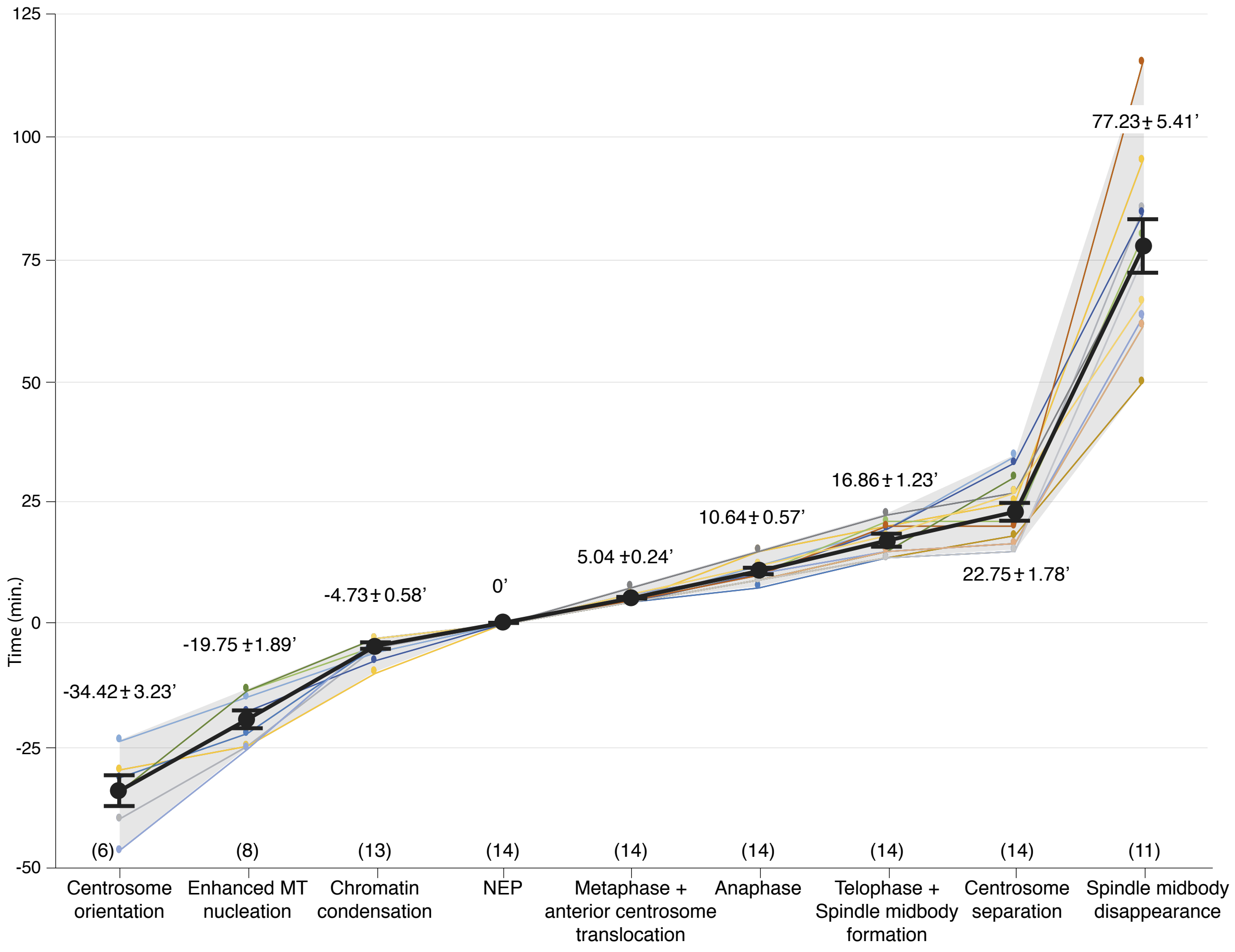

Fig. S4. Quantification of cellular events during GSC mitosis. We quantified nine events that took place during the mitosis of 14 live GSCs. Not all of the events were captured in the movies for the entire set of GSCs. Data for each of the GSCs analysed is colour coded, while the thick, black line represents average values for all of the events. Sample size (n) corresponds to GSCs analysed for each event. The starting time of each of the events (mean ± SEM) is shown. Related to Figure 5.

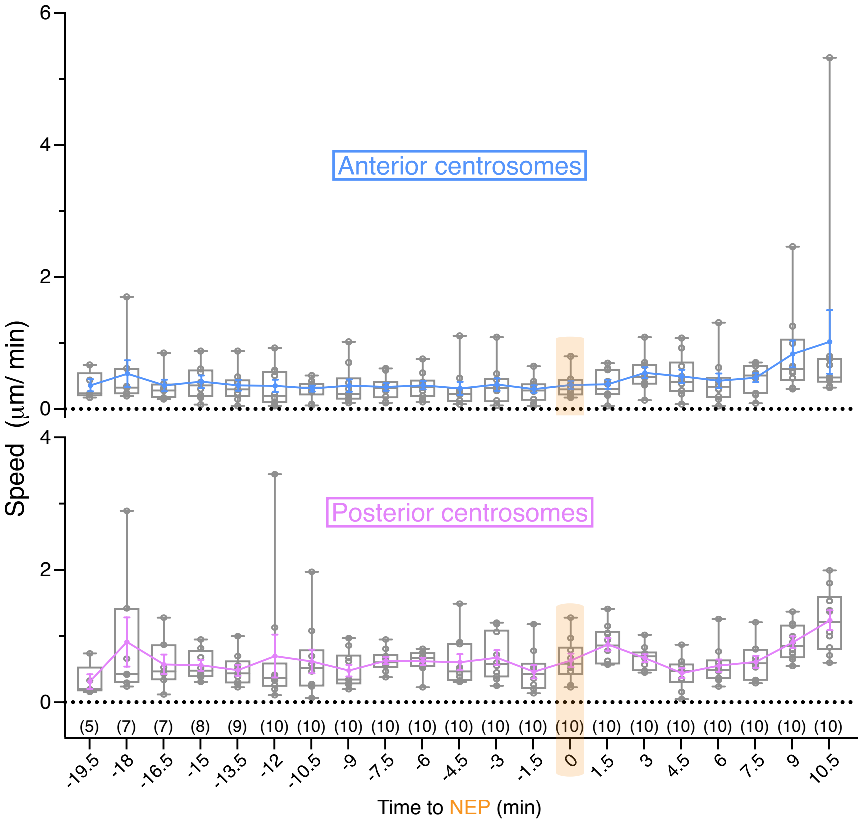

Anterior centrosomes
Posterior centrosomes
Speed (μm/ min)
6
4
2
0
4
2
0
(5) (7) (7) (8) (9) (10) (10) (10) (10) (10) (10) (10) (10) (10) (10) (10) (10) (10) (10) (10) (10)
-19.5 -18 -16.5 -15 -13.5 -12 -10.5 -9 -7.5 -6 -4.5 -3 -1.5 0 1.5 3 4.5 6 7.5 9 10.5
Time to NEP (min)

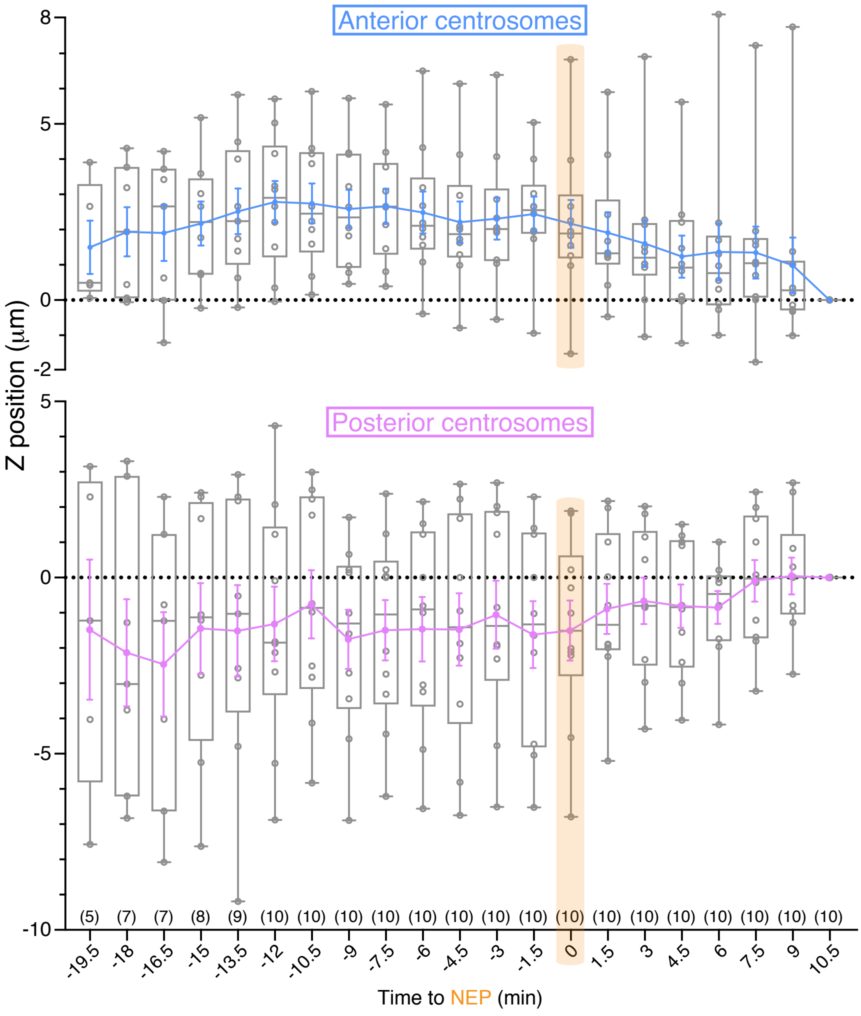

Anterior centrosomes
Posterior centrosomes
Z position (μm)
8
5
0
-2
5
0
-5
-10
(5) (7) (7) (8) (9) (10) (10) (10) (10) (10) (10) (10) (10) (10) (10) (10) (10) (10) (10) (10) (10)
-19.5 -18 -16.5 -15 -13.5 -12 -10.5 -9 -7.5 -6 -4.5 -3 -1.5 0 1.5 3 4.5 6 7.5 9 10.5
Time to NEP (min)

Fig. S5. Quantification of the speeds and Z positions of anterior and posterior GSC centrosomes before metaphase. We quantified the anterior and posterior centrosome movements of ten GSCs prior to and after NEP (t= 0’). The individual values of each of the measurements taken are represented in the box and whisker graphs. In the Z position graphs, the 0 line indicates the final position of the centrosome at time t= 10.5’. We assigned a positive value to the t= -19.5’ position of the anterior centrosomes, and this set the reference for the position of the sibling posterior centrosomes. The coloured lines represent the MEAN; error bars correspond to the SEM. Digits in parenthesis refer to the number of centrosomes analysed in each of the 21 time points. Related to Movie S14 and Fig. S5.

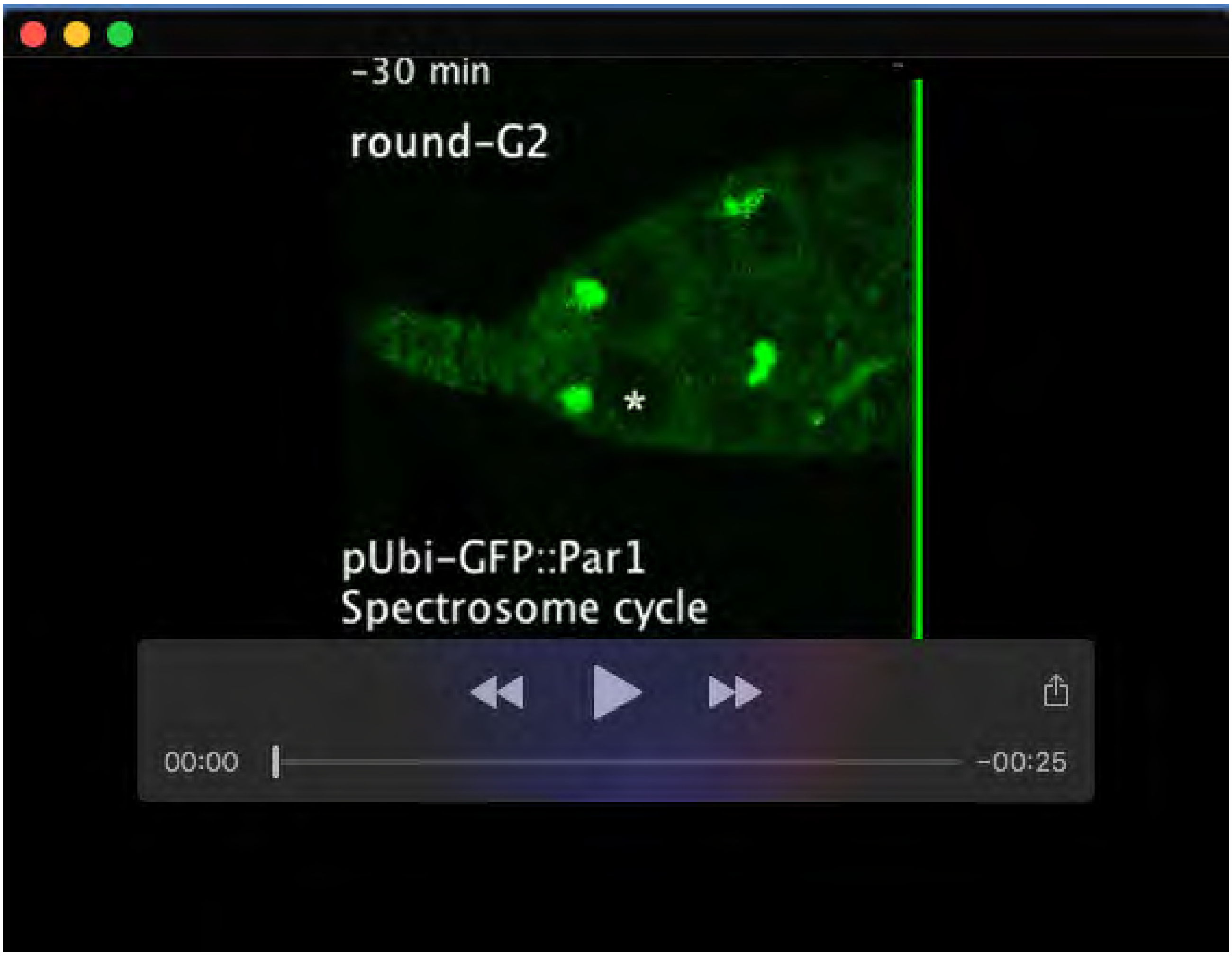

Movie 1. Spectrosome cycle in live GSCs. 740-minute-long time-lapse movie showing the changes in spectrosome morphology of a *GFP::par-1* germarium. The spectrosome morphologies observed are indicated. Asterisk: GSC; CB: cystoblast. The movie is a maximum projection of 4-7 z-planes (1 μm each) per time point (taken every 10 minutes). t= 0' corresponds to NEP. Related to Fig. 1B.

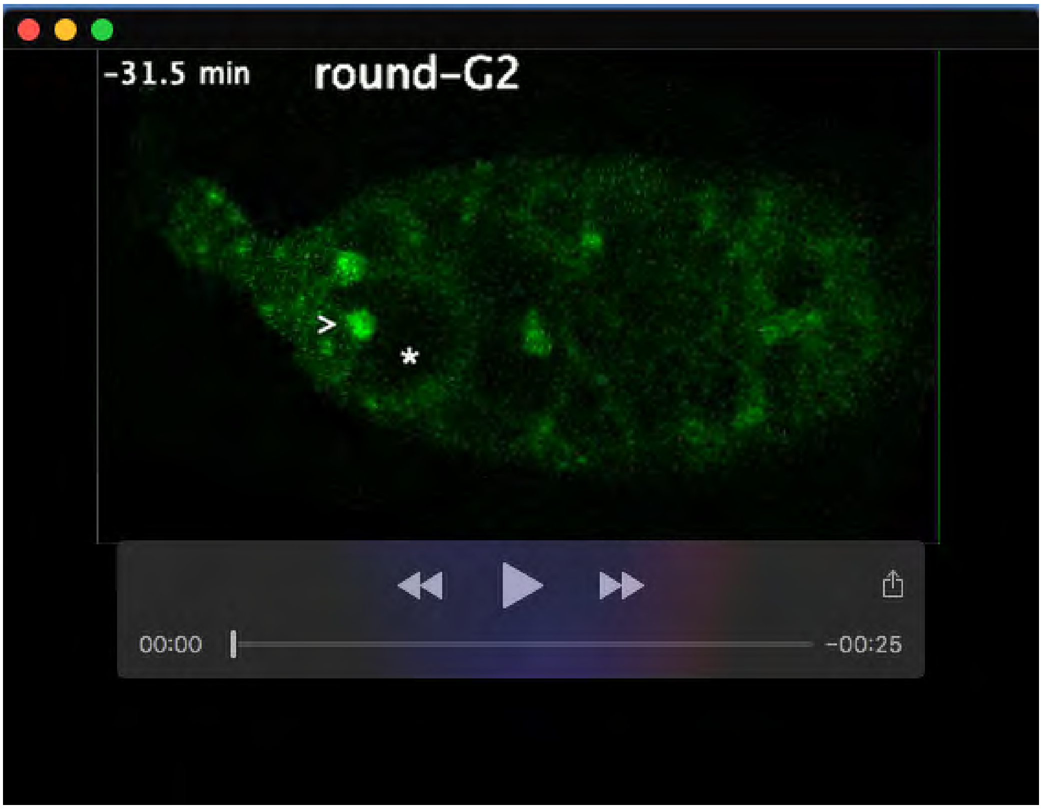

Movie 2. GFP::Par-1 is released from the GSC spectrosome during mitosis. 70.5-minute-long time-lapse movie showing that GFP::Par-1 is released from the spectrosome to the cytoplasm at t= -6'. Asterisk: GSC; CB: cystoblast; white open arrow: anterior spectrosome; yellow open arrow: spectrosome plug between the GSC and the CB. The movie is a maximum projection of 5-9 z-planes (1 µm each) per time point (taken every 1.5 minutes). t= 0' corresponds to NEP. Related to Fig. 2A and Fig. S1D.

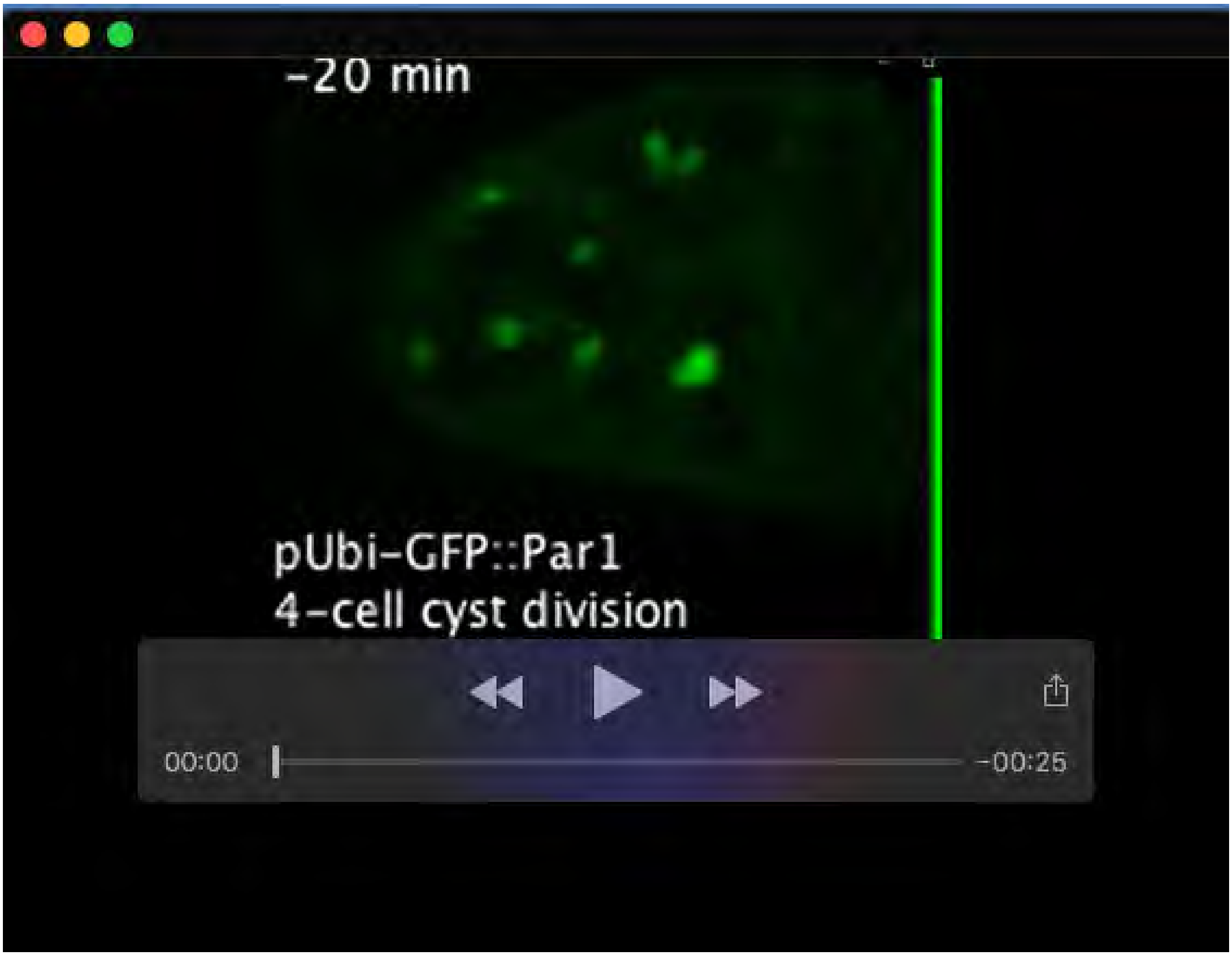

Movie 3. GFP::Par-1 is released from 4-cell-cyst fusomes during mitosis. A 190-minute-long time-lapse movie showing that GFP::Par-1 is released from the fusome of a 4-cell-cyst to the cytoplasm at t= -10’. White line: 4-cell cyst; white open arrow: fusome plugs. The movie is a maximum projection of 5-9 z-planes (1 µm each) per time point (taken every 10 minutes). t= 0’ corresponds to the onset of mitosis. Related to Fig. 2A.

Development • Supplementary information

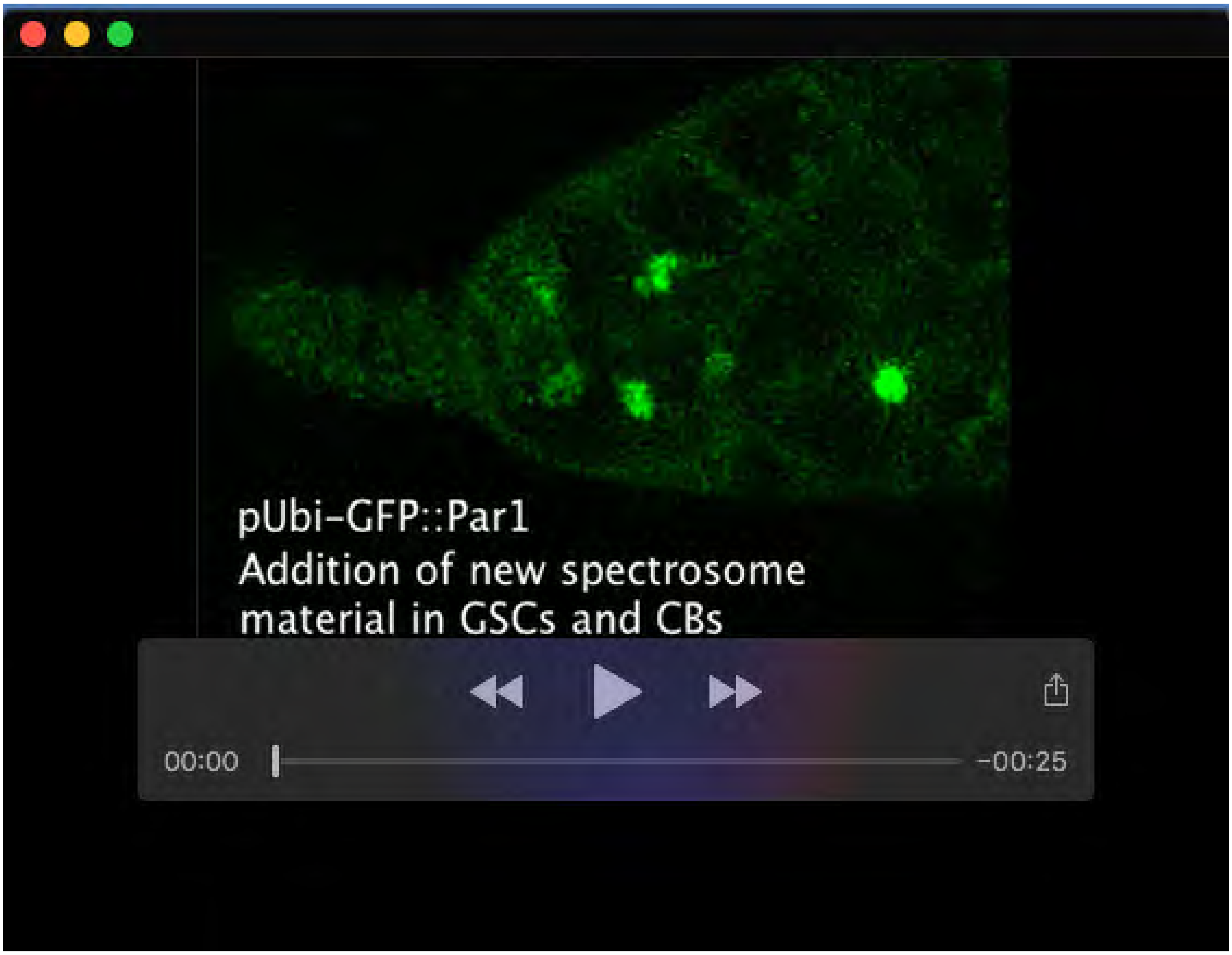

Movie 4. Addition of new spectrosome material in GSCs and CBs. 210-minute-long time-lapse movie showing the incorporation of new GFP::Par-1-positive material into the GSC- (yellow open arrows) and CB- (white open arrows) sides of the equatorial plug. The movie is a maximum projection of 5-9 z-planes (1 µm each) per time point (taken every 10 minutes). Related to Fig. 2B, C.

Development • Supplementary information

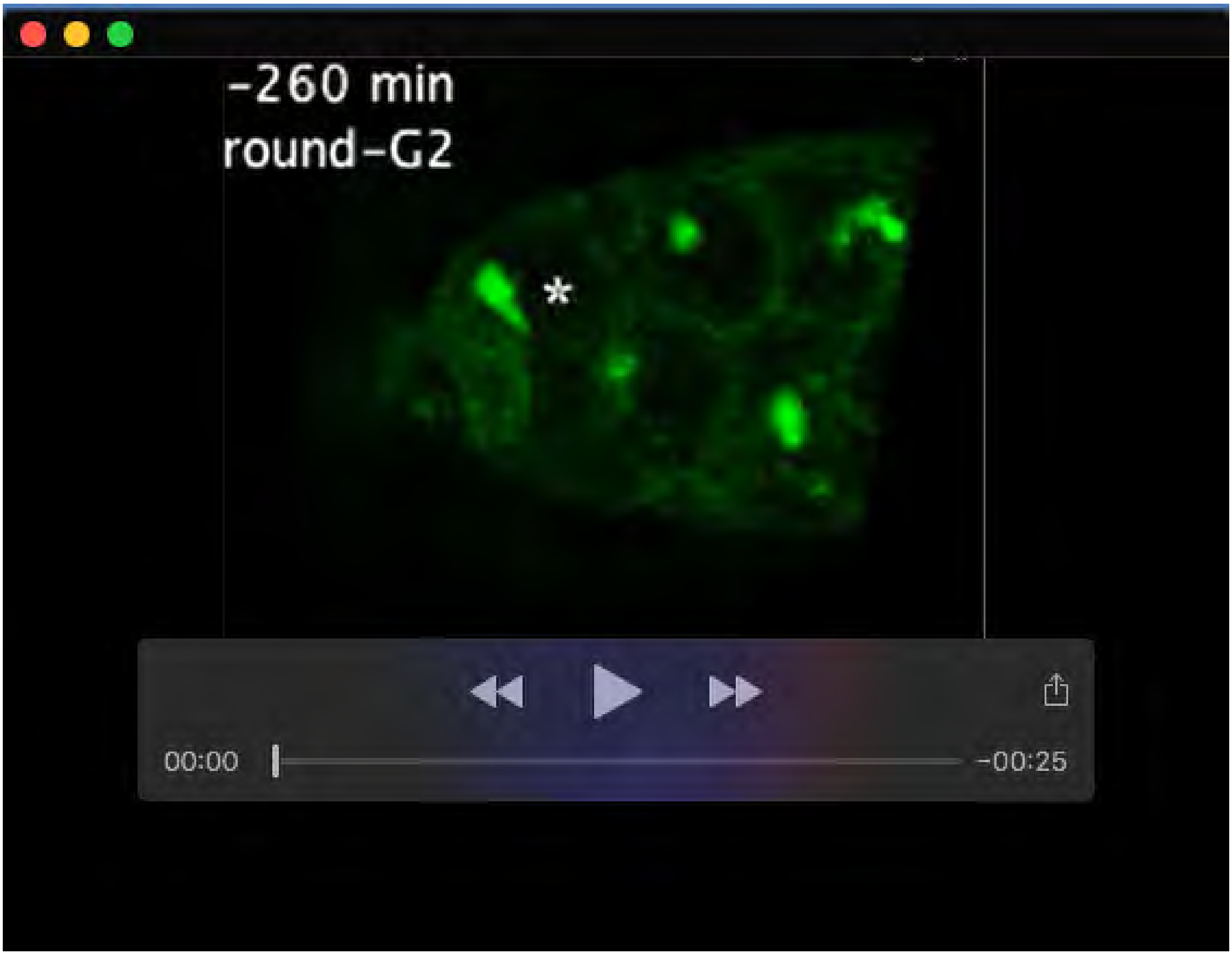

Movie 5. Addition of new material to a "round-G2" spectrosome. 290-minute-long time-lapse movie showing the incorporation of new GFP::Par-1-positive material into a "round-G2" spectrosome (white open arrow) even until few minutes prior to mitosis. Asterisk: GSC; CB: cystoblast. The movie is a maximum projection of 5-9 z-planes (1 µm each) per time point (taken every 10 minutes). t= 0' corresponds to NEP. Related to Fig. S2A.

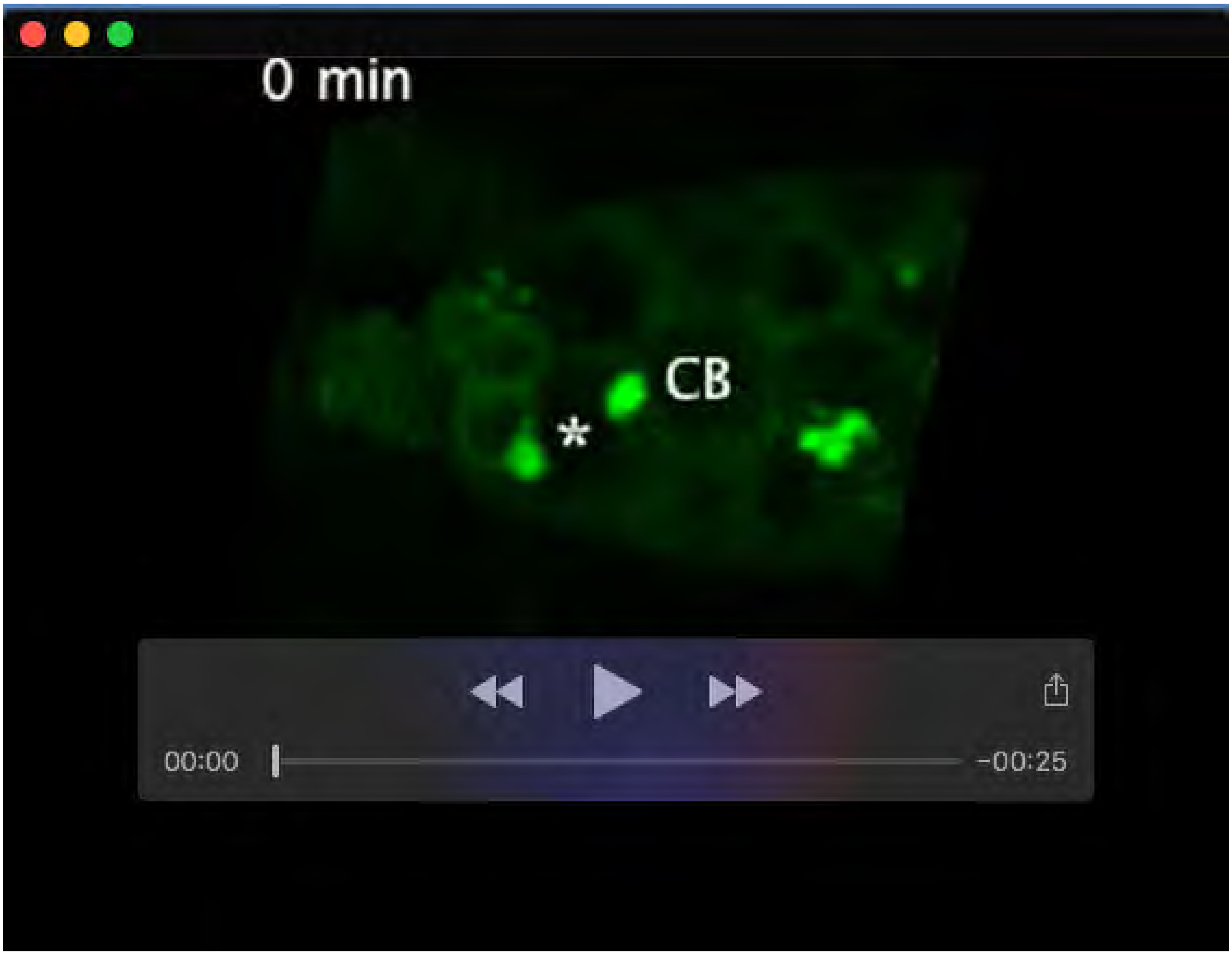

Movie 6. Addition of new spectrosome material in CBs. 480-minute-long time-lapse movie showing the incorporation of new GFP::Par-1-positive material into the CB spectrosome (white open arrows). Most of the new material is generated in the posterior half of the cell (t= 170' to 440'). Note the large lump of new material that starts appearing at t= 290' and that will eventually fuse at the posterior with the drifting CB spectrosome that results from the abscission of the GSC/CB intercellular bridge (t= 460'). Asterisk: GSC; CB: cystoblast. The movie is a maximum projection of 5-9 z-planes (1 μm each) per time point (taken every 10 minutes). While this germarium is the same as the one shown in Movie S9, the z-planes selected to compose this movie are different. Related to Fig. 2C.

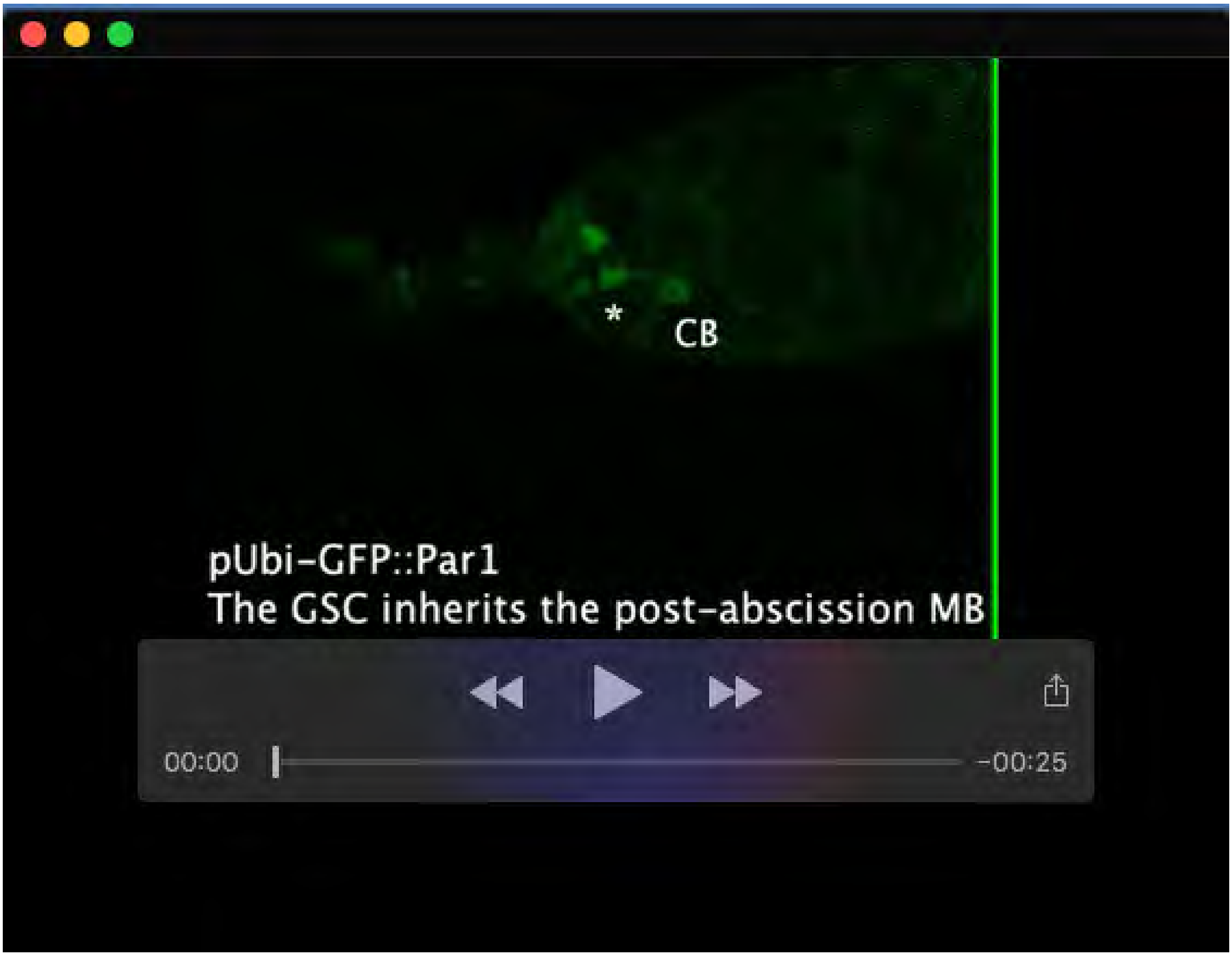

Movie 7. The GSC inherits the post-abscission midbody. 380-minute-long time-lapse movie of a *GFP::par-1* germarium showing that the post-abscission midbody (MB) is inherited by the GSC and that it fuses with the anterior spectrosome after moving towards the GSC/CpC interface (t= 330'). Asterisk: GSC; CB: cystoblast. The movie is a maximum projection of 5-9 z-planes (1 µm each) per time point (taken every 10 minutes). Related to Fig. 2D.

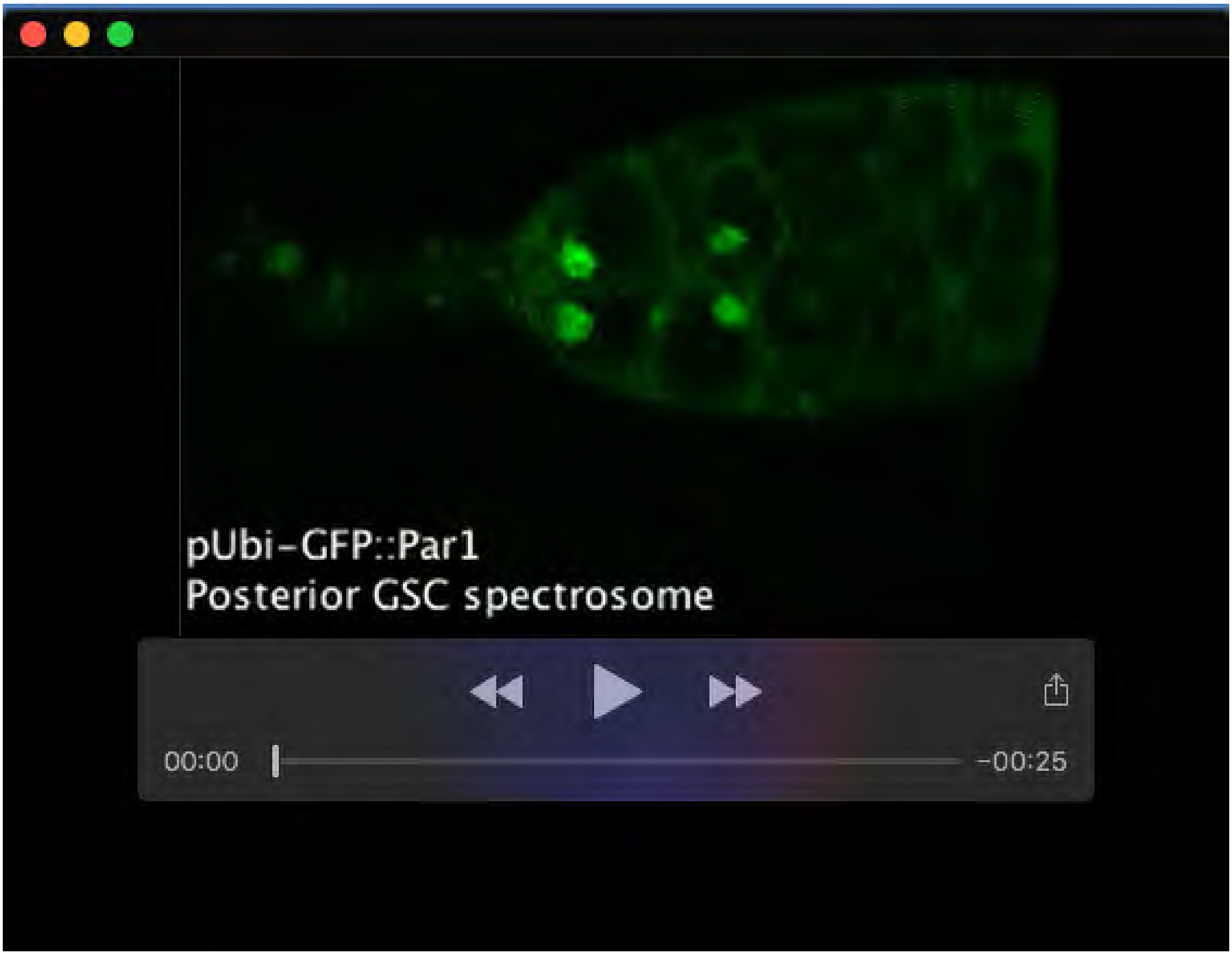

Movie 8. Formation of a posterior GSC spectrosome. 420-minute-long time-lapse movie of a *GFP::par-1* germarium showing a mitotic GSC in which the spectrosome detaches from the GSC/CpC interface (t= 20') and moves to the posterior until it fuses with the equatorial plug (t= 100'). White asterisk: GSC; white open arrow: GSC spectrosome; CB: cystoblast; purple asterisks: cap cells; white arrow: "scar" of anterior spectrosome material. The movie is a maximum projection of 5-9 z-planes (1 µm each) per time point (taken every 10 minutes). Related to Fig. S3.

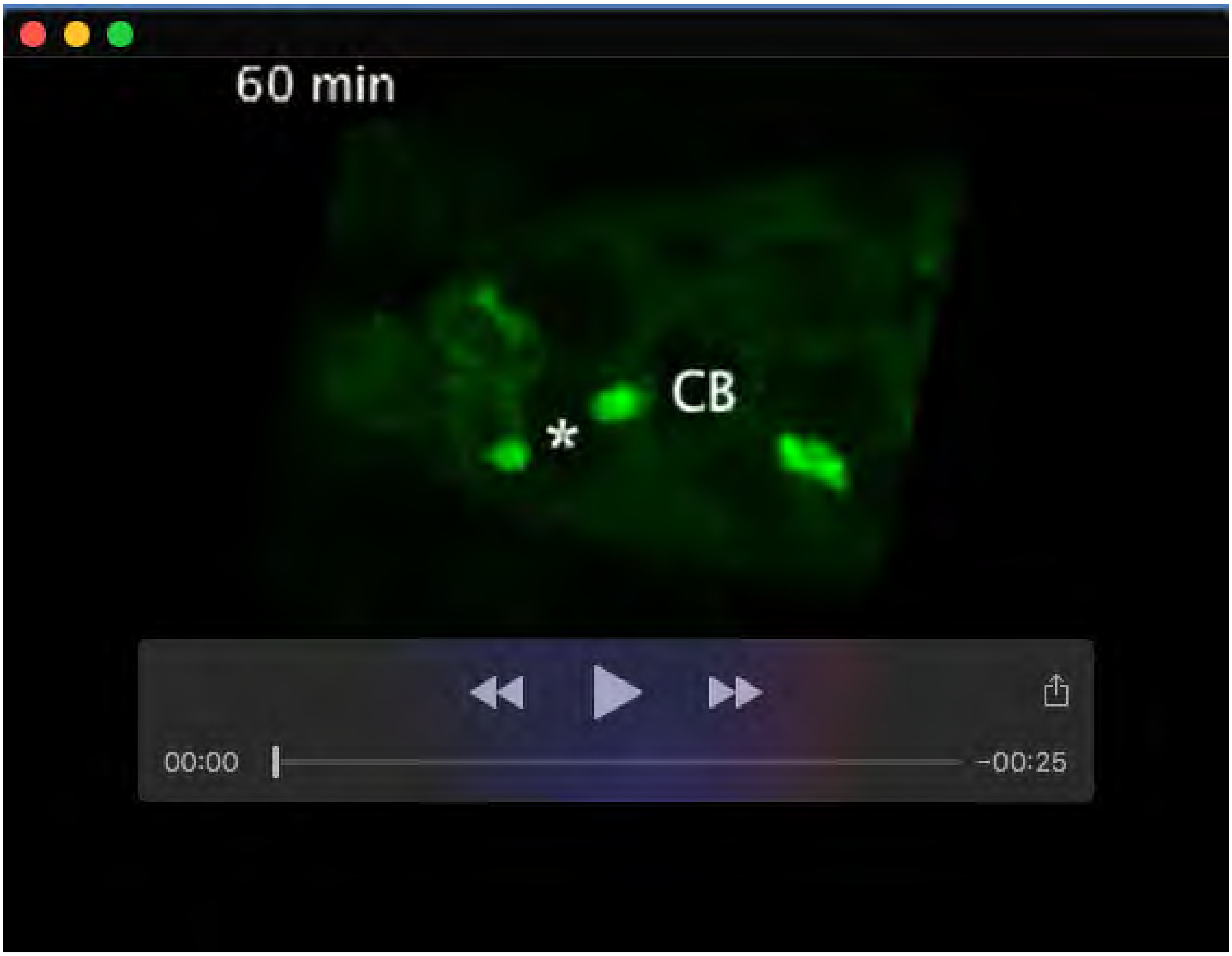

Movie 9. Abscission of a posterior GSC spectrosome followed by its anterior re-positioning. 460-minute-long time-lapse movie of a *GFP::par-1* germarium showing a posterior spectrosome during cytokinesis (t= 120' to 140'), the subsequent formation of a post-abscission midbody (t= 150') and the separation of GSC and CB spectrosomes (t= 240'). The GSC spectrosome then moves towards the GSC/CpC interface to fuse with the anterior portion of the spectrosome (t= 480'). Asterisk: GSC; CB: cystoblast; white open arrow: post-abscission midbody. The movie is a maximum projection of 4-9 z-planes (1 μm each) per time point (taken every 10 minutes). While this germarium is the same as the one shown in Movie S6, the z-planes selected to compose this movie are different. Related to Fig. S3.

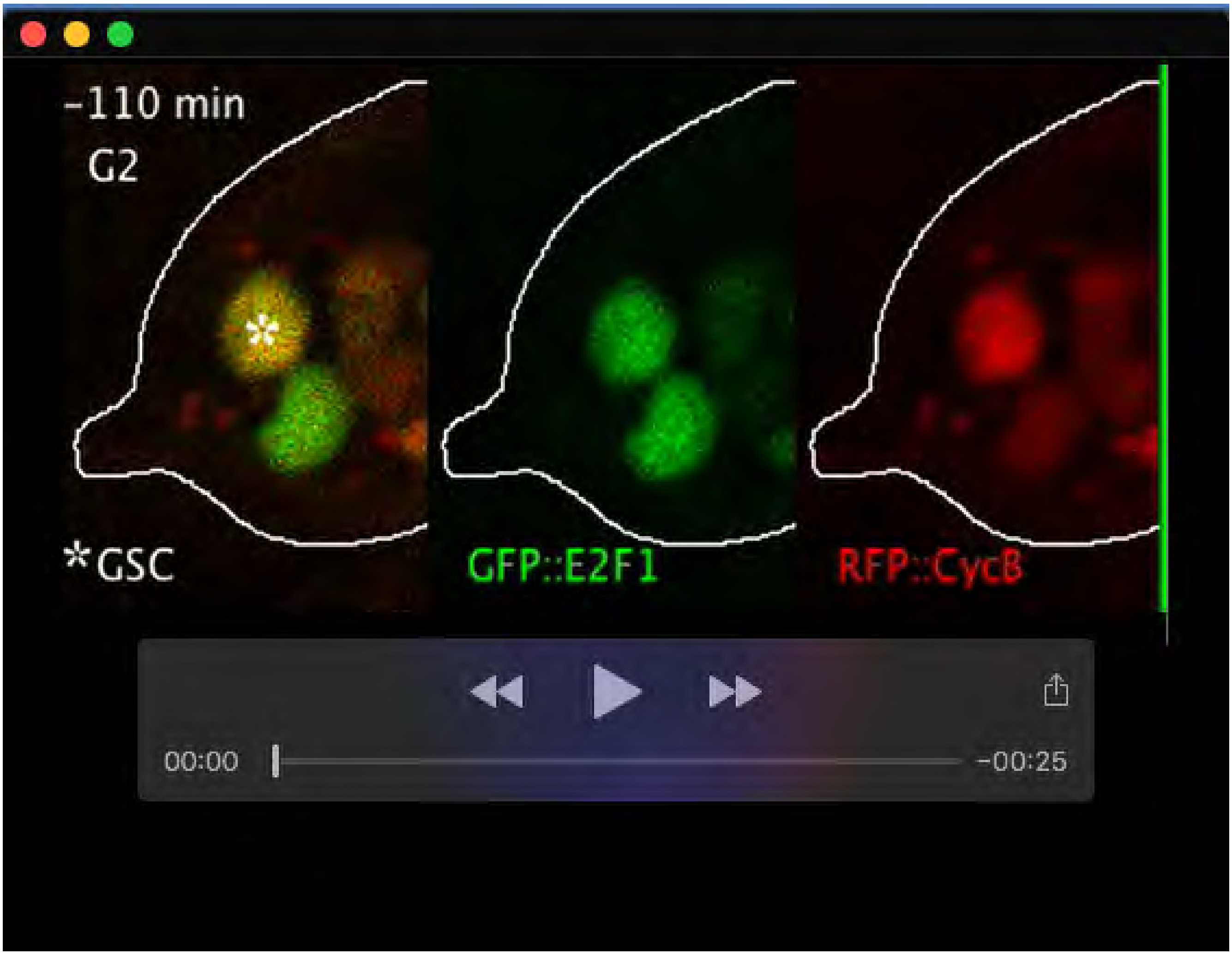

Movie 10. Expression of Fly-FUCCI markers in a live GSC. 500-minute-long time-lapse movie of a *nanos>GFP::E2f1 + mRFP1::nls-CycB* germarium showing the distribution of the Fly-FUCCI markers throughout the cell cycle. In G2 (t= -110’ to -10’), the cell expresses both GFP- and RFP-fusions. At NEP, both protein fusions are released into the cytoplasm (t= 0’). At the M/G1 transition, the RFP signal disappears and only the GFP signal is detected (t= 20’). From t= 30’ to 130’, neither GFP nor RFP are visible. At the onset of S phase (t= 140’), the RFP fusion is detected. At the S/G2 transition, the GFP fusion begins to be visible (t= 210’). Asterisk: dividing GSC. The movie is a maximum projection of 5-9 z-planes (1 μm each) per time point (taken every 10 minutes). Related to Fig. 3.

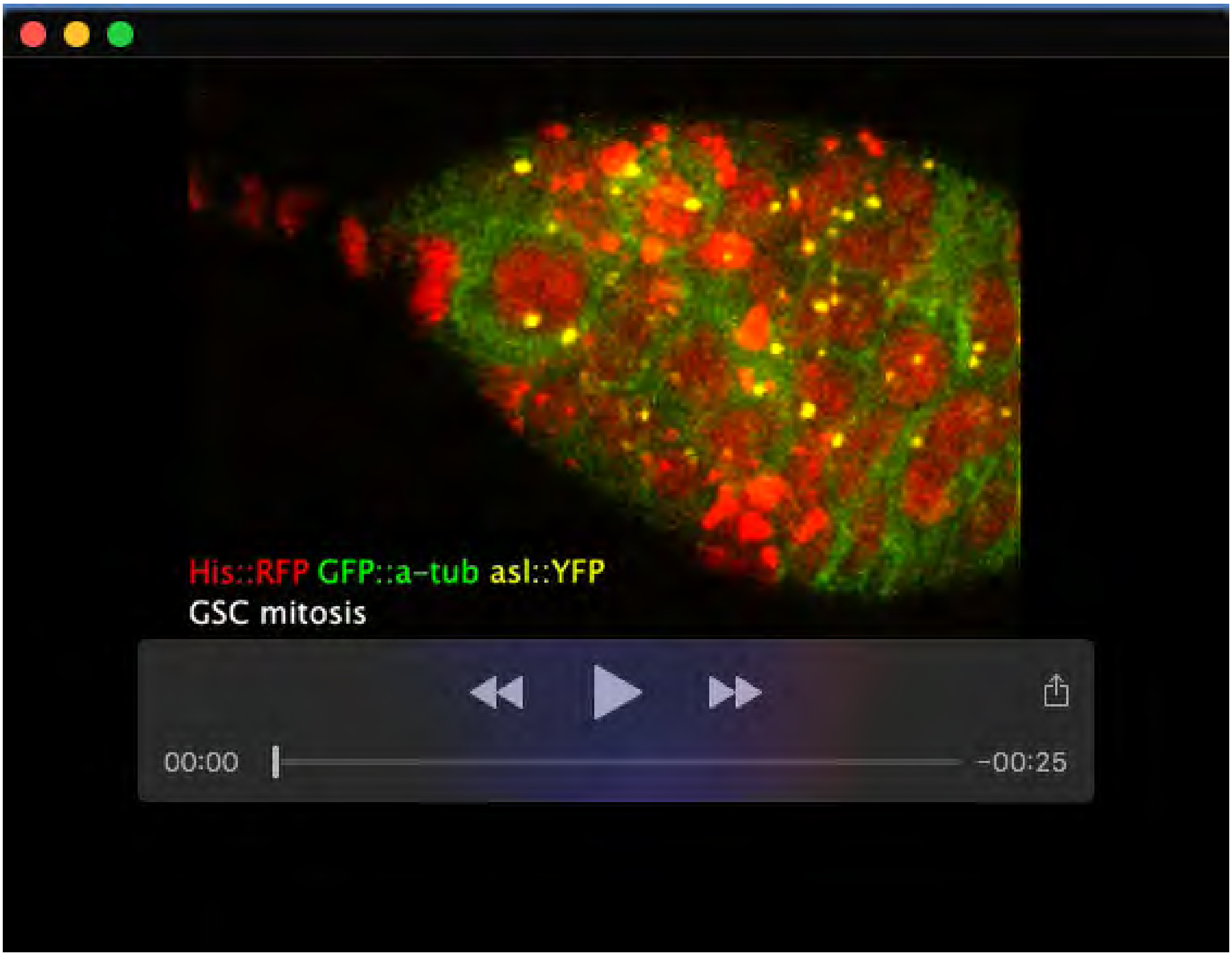

Movie 11. Cellular events during GSC mitosis. 129-minute-long time-lapse movie of a *YFP::asl, GFP::αtubulin, his::RFP* germarium to label centrosomes, microtubules and chromatin, respectively, during a GSC division. The orientation of centrosomes (t= -30'), increased microtubule nucleation (t= -15'), chromatin condensation (t= -6'), NEP (t= -0'), metaphase (t= 3.5' to 10.50'), centrosome translocation to the cell cortex (t= 4.5' to 7.5'), anaphase (t= 12' to 13.5') and telophase + spindle midbody formation (t= 15' to 18') are indicated. Asterisk: dividing GSC; CB: cystoblast; blue open arrowhead: anterior centrosome; yellow open arrowhead: posterior centrosome. The movie is a maximum projection of 4-9 z-planes (1 μm each) per time point (taken every 1.5 minutes). Related to Fig. 5A.

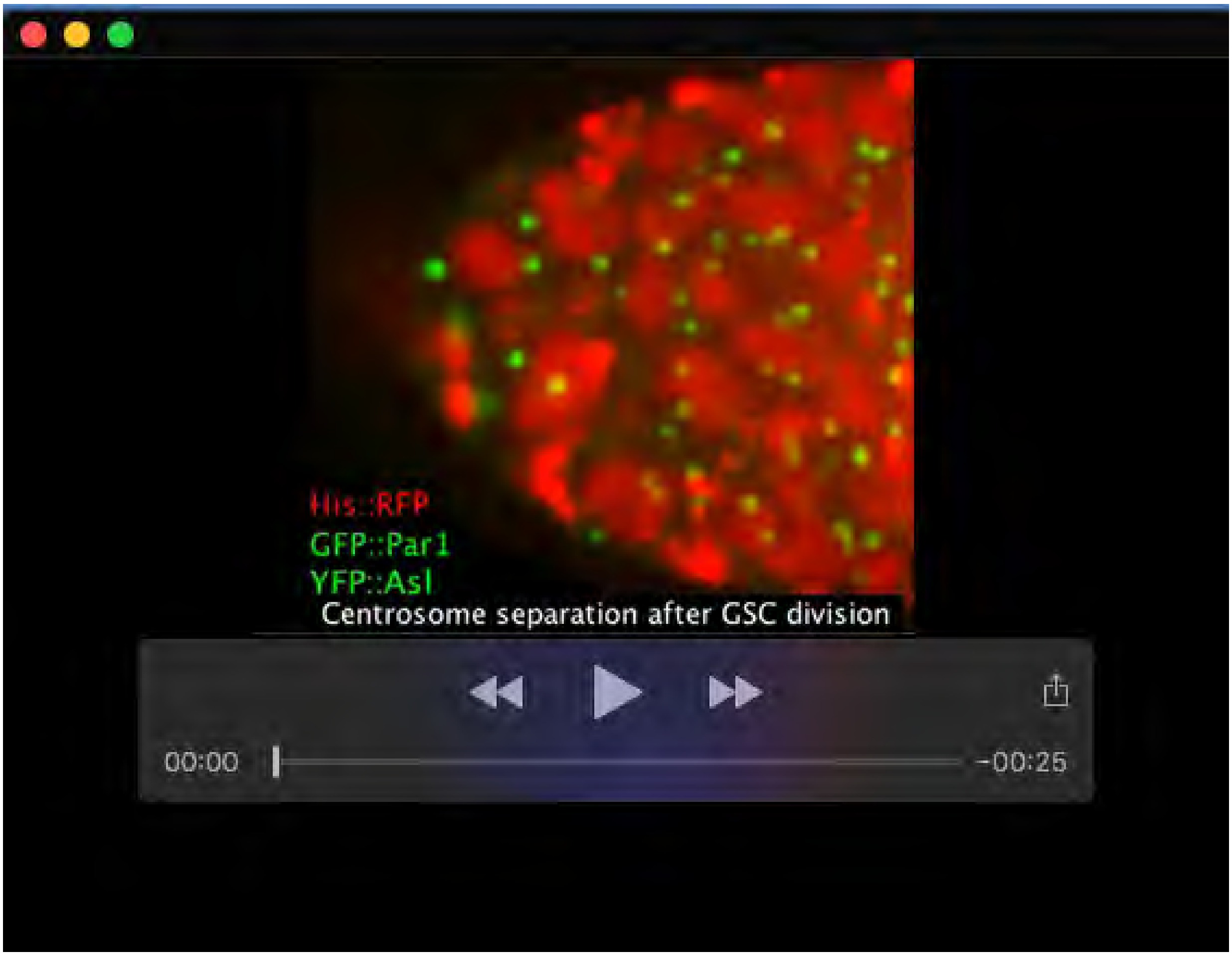

Movie 12. The anterior centrosome associates with the spectrosome in mitosis. Centrosome separation occurs early in G1. 75-minute time-lapse movie of a *GFP::asl, GFP::par-1, his::RFP* germarium showing the association of the GSC anterior centrosome with the spectrosome at t= -6'. Subsequently, the centrosome translocates to the GSC cortex adjacent to the cap cells (t= 6'). At t= 36', the centrosome splits and two GFP::Asl-positive dots are observed. Note that the sibling CB also undergoes centrosome separation soon after the GSC event (t= 43.5'). t= 0' corresponds to NEP. Yellow arrow: GSC spectrosome; yellow open arrowheads: GSC centrosomes; CB: cystoblast; orange open arrowheads: CB centrosomes. The movie is a maximum projection of 5-9 z-planes (1 µm each) per time point (taken every 1.5 minutes). Related to Fig. 5B.

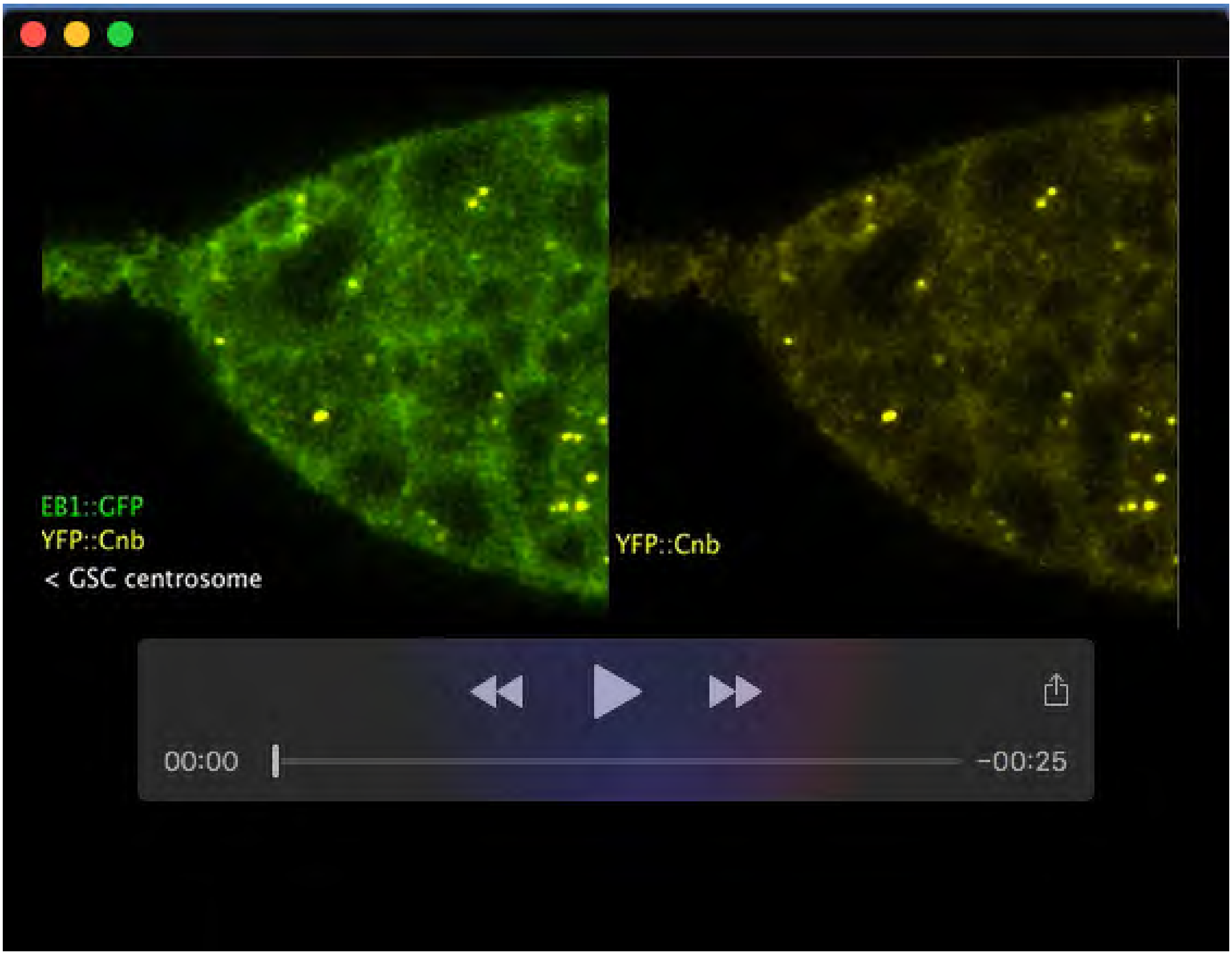

Movie 13. GSC centrosomes separate before centriole duplication. 121-minute time-lapse movie of an *EB1::GFP, YFP::cnb* germarium to label nucleating microtubules and daughter centrioles, respectively. The movie shows a GSC that, upon division, contains only one YFP::Cnb-positive dot, at least until 108’ after NEP. White open arrowheads: GSC centrosomes. The movie is a maximum projection of 5-9 z-planes (1 µm each) per time point (taken every 1.5 minutes). Related to Fig. 6A.

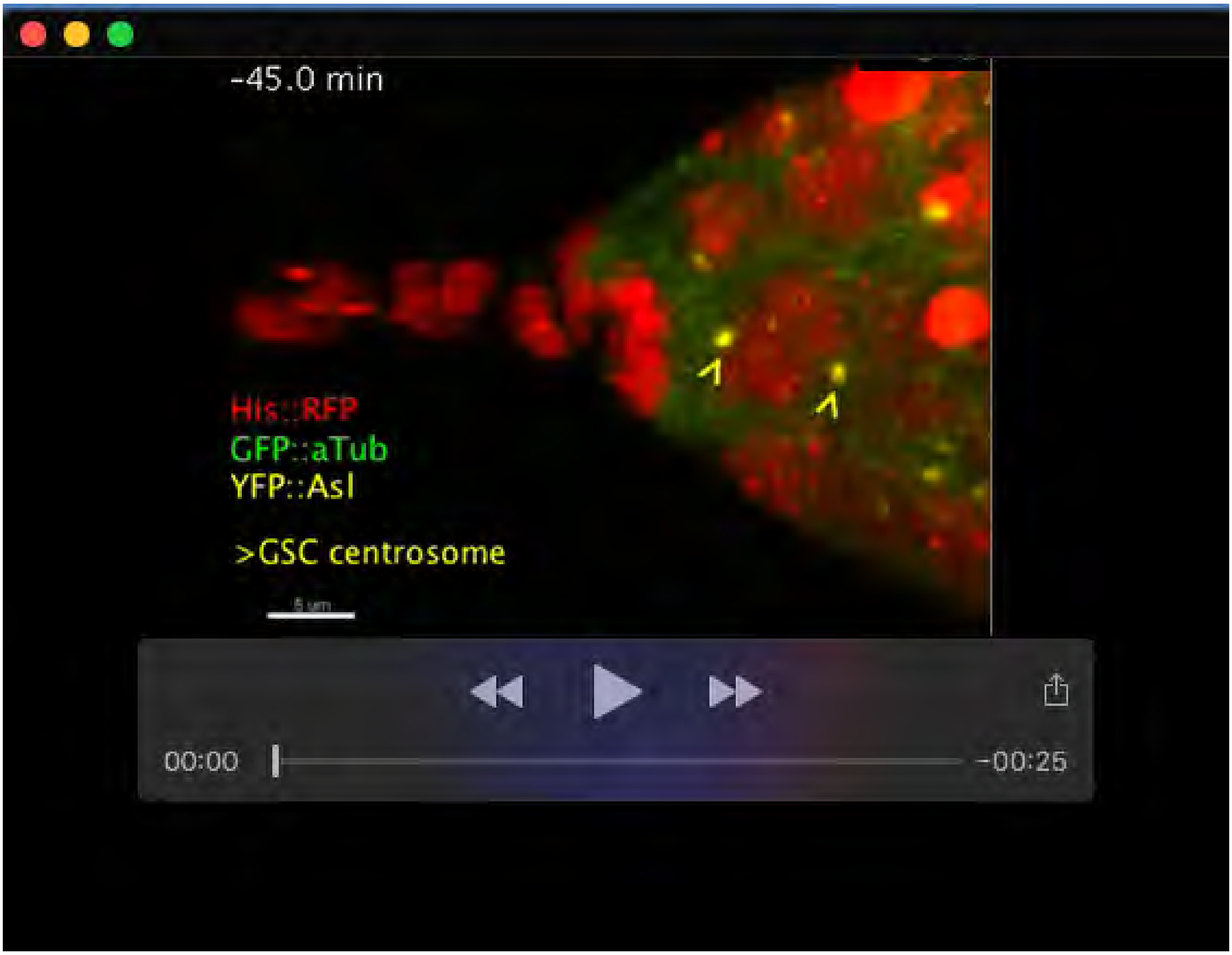

Movie 14. Tracking of GSC centrosomes 30' prior to metaphase plate formation. Combination of a 3D rendition of a *YFP::asl, GFP::α-Tub, his::RFP* germarium and the software-generated tracking of the movements of the GSC centrosomes 30' before the formation of the metaphase plate. Related to Fig. 7 and Fig. S5.

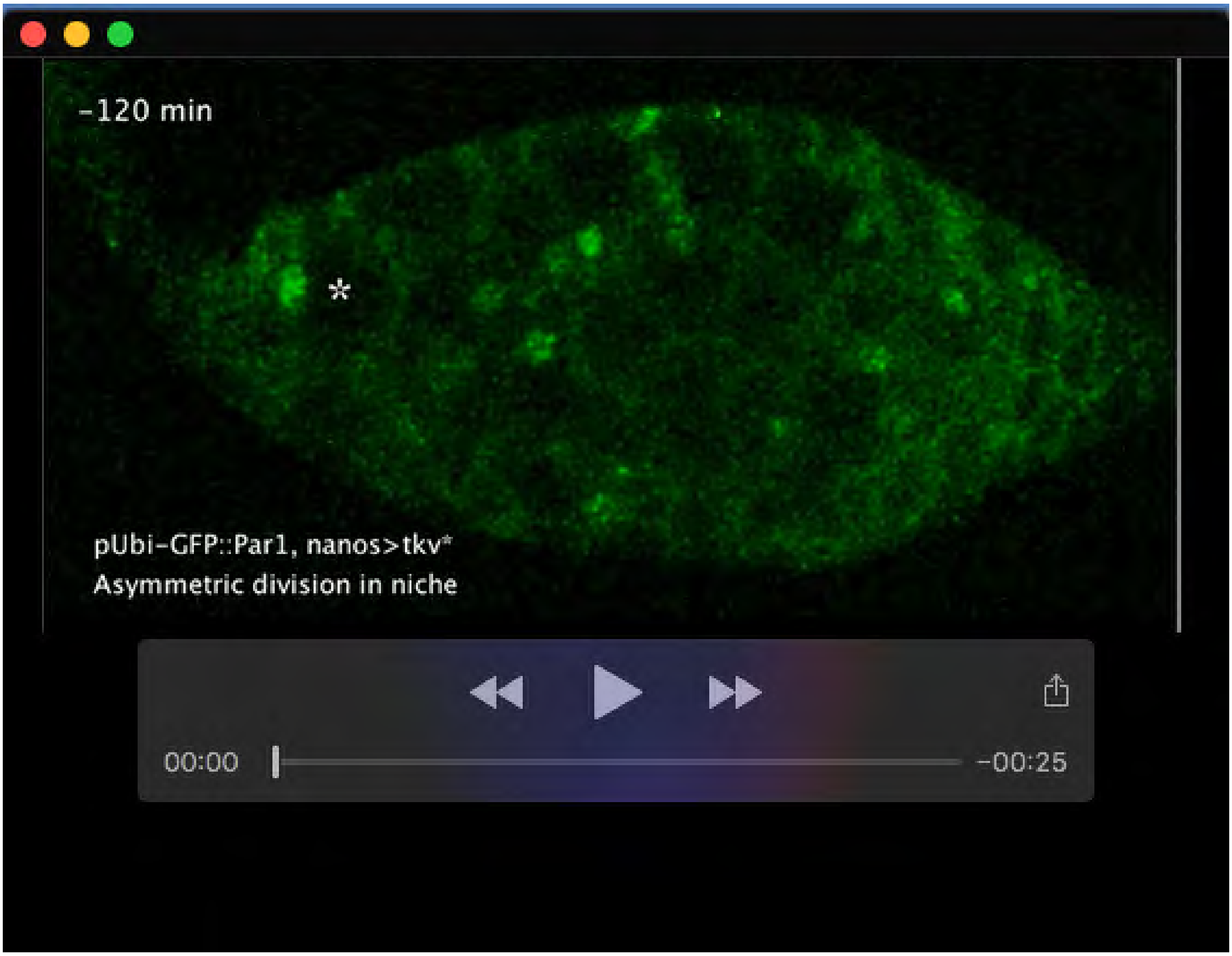

Movie 15. Division of a GSC adjacent to the CpC rosette in a tumorous germarium.

850-minute-long time-lapse movie showing the asymmetric division of a GSC located next to the cap cells in a *GFP::par-1*, *nanos>tkv^{Act}* germarium. The spectrosome morphologies observed are indicated. Asterisk: GSC; DC: daughter cell (as this is a *nanos>tkv^{Act}* germarium, we cannot consider the daughter of a GSC a cystoblast). The movie is a maximum projection of 3-7 z-planes (1 μm each) per time point (taken every 10 minutes). t= 0' corresponds to NEP. Related to Fig. 8B.

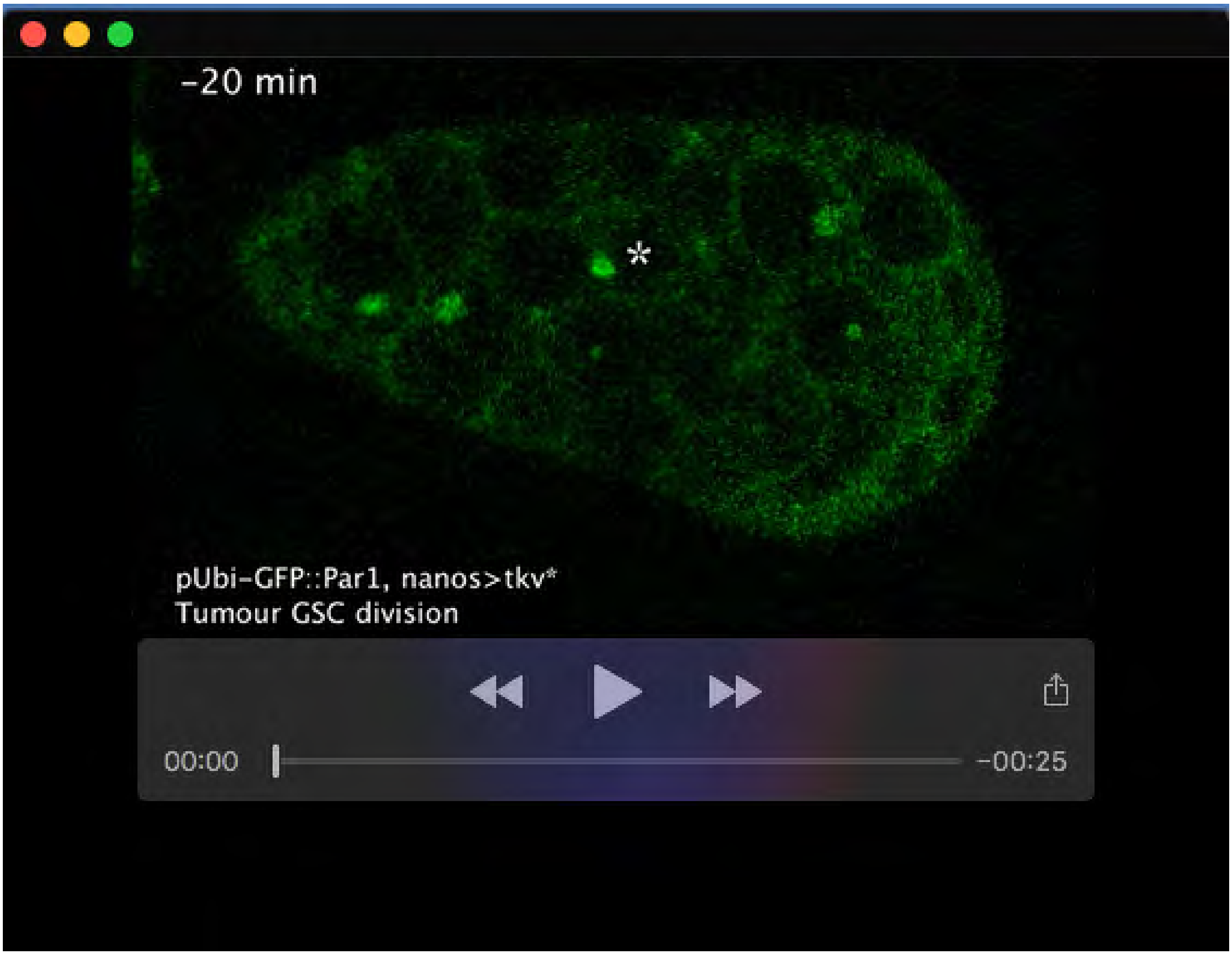

Movie 16. Division and cytokinesis of GSC-like tumour cells. 360- and 570-minute time-lapse movies of *GFP::par-1*, *nanos>tkv*^{Act} germaria showing the division of a GSC-like tumour cell in which the spectrosome translocates from one pole of the cell, opposite to the future cytokinetic ring, to the bridge connecting both cells upon division. The spectrosome then grows symmetrically between both daughter cells (Movie 1). Movie 2 captures the cytokinesis of a pair of interconnected cells followed by their seemingly random movement. Movie 1- asterisks: mitotic GSC-like tumour cell and its daughters; white open arrowhead: GSC-like spectrosome and new spectrosome material; yellow open arrowhead: GFP::Par-1-positive plug filling the intercellular bridge connecting both daughter cells. Movie 2- white arrowhead: spectrosome at the intercellular bridge and in the daughter cells; white open arrowhead: post-abscission midbody. Related to Fig. 8C, D.
