# Supplementary material for: Live imaging of the Drosophila ovarian niche shows spectrosome and centrosome dynamics during asymmetric germline stem cell division
Source: Development. 2021 Sep 17;148(18):dev199716. doi: 10.1242/dev.199716 (PMC8489027; doi:10.1242/dev.199716)
Supplement: Reviewer comments [file develop-148-199716_review_history.pdf]

Live imaging of the *Drosophila* ovarian niche shows spectrosome and centrosome dynamics during asymmetric germline stem cell division

Gema Villa-Fombuena, María Lobo-Pecellín, Miriam Marín-Menguiano, Patricia Rojas-Ríos and Acaimo González-Reyes
DOI: 10.1242/dev.199716

Editor: Cassandra Extavour

Review timeline

Original submission:	19 April 2021
Editorial decision:	7 June 2021
First revision received:	7 July 2021
Accepted:	29 July 2021

Original submission

First decision letter

MS ID#: DEVELOP/2021/199716

MS TITLE: Live imaging of the Drosophila ovarian niche shows spectrosome and centrosome dynamics during asymmetric germline stem cell division

AUTHORS: Gema Villa-Fombuena, Maria Lobo-Pecellin, Miriam Marin-Menguiano, Patricia Rojas-Rios, and Acaimo Gonzalez-Reyes

I have now received all the referees reports on the above manuscript, and have reached a decision. The referees' comments are appended below, or you can access them online: please go to BenchPress and click on the 'Manuscripts with Decisions' queue in the Author Area.

The overall evaluation is positive and we would like to publish a revised manuscript in Development, provided that the referees' comments can be satisfactorily addressed. Please attend to all of the reviewers' comments in your revised manuscript and detail them in your point-by-point response. If you do not agree with any of their criticisms or suggestions explain clearly why this is so.

We are aware that you may currently be unable to access the lab to undertake experimental revisions. If it would be helpful, we encourage you to contact us to discuss your revision in greater detail. Please send us a point-by-point response indicating where you are able to address concerns raised (either experimentally or by changes to the text) and where you will not be able to do so within the normal timeframe of a revision. We will then provide further guidance. Please also note that we are happy to extend revision timeframes as necessary.

Reviewer 1

Advance summary and potential significance to field

The authors have used live imaging of ex vivo cultured germaria to characterize the dynamics of GSC division. By using transgenic reporters that label components important for cell division, Villa-

© 2021. Published by The Company of Biologists under the terms of the Creative Commons Attribution License (https://creativecommons.org/licenses/by/4.0/).

Fombuena and colleagues quantify duration of cell cycle phases, correlate spectrosome morphology with GSC cell cycle phase examine GSC centrosome behavior before mitosis, and assess division of GSC-like cells in germline tumors. While the spectrosome morphological changes during the cell cycle have been previously described, the work discussed in this manuscript advances the field by carefully detailing morphological and temporal spectrosome and centrosome dynamics during GSC division. The manuscript is well written and experiments have been carefully executed but the novelty and implications for GSC/stem cell behavior are modest.

Comments for the author

Line-by-line comments

Major issues

Lines 96-98: Live imaging of the germaria has been previously described (Morris and Spradling, 2011; Valencia-Exposito, et al., 2016). While the authors mention that they have “defined experimental conditions that allow prolonged observations of ex vivo cultured germaria”, this is not a major part of the main text; instead it has been relegated to the methods and supplementary data. If development of these conditions is a major part of being able to live image spectrosome behavior the authors should consider making this a component of the main text. In addition the implications of the work might be more obvious if the authors highlight how this tool can be used to analyze other aspects of oogenesis.

Lines 157-158: It is not clear if development of the long-term ex vivo imaging system included a test of various time intervals. If so, it is worth mentioning to further support using the 10-minute interval. If not, a test of shorter imaging time intervals might identify a time interval that allows more frequent sampling for better time resolution.

In many cases, the authors do not indicate what percentage of GSCs analyzed display the pattern described. For example: lines 180-183, lines 203-206, lines 250-251, line 341, line 352, lines 414-417, Lines 414-417: How often did GSCs deviate from this “canonical” pattern? Was there another pattern observed?

Regarding statistical analysis, no data presented were quantified in such a way that statistical analysis could be conducted. Therefore, the “Statistical Analysis” section of Materials and Methods should be renamed to more accurately match what is being described.
Minor Issues Line 75: Change the “or” in “serine-threonine kinase Par-1 or the membrane skeletal” to “and”

Lines 77-80: Add in the additional references
Lines 92-95: There are additional references for this. For example, Dpp overexpression data shown in Xie and Spradling, 1998
Line 99: “stablish” should be “establish”
Lines 119-121: Since the change in GFP signal is very subtle, quantification may be a good way to exemplify this.
Line 123-124: Indicate how many germaria this corresponds to.
Line 143-144: Add references
Line 147: “begun” should be “began” or “begins”
Line 171: “publish” should be “published”
Lines 180-183: It would be nice to see a quantification of Par1::GFP nucleoplasm intensity over each timepoint.
Line 211: Blob word choice – maybe mass, or globule, etc
Line 251: “Of the 23 GSCs” – over how many germaria?
Line 292: Instead of “black”, “lacking label”, or something to that effect
Line 384: Instead of “We could observed”, “We observed” or “We could observe”
Lines 454-456: not sure what this part of the sentence is supposed to say

<u>Reviewer 2</u>

© 2021. Published by The Company of Biologists under the terms of the Creative Commons Attribution License (https://creativecommons.org/licenses/by/4.0/).

Advance summary and potential significance to field

In this study, the authors combined short and long-term ex vivo live imaging with fluorescently tagged proteins to characterize the cell cycle of Drosophila germline stem cells. Their observations provide strong confirmation of previous conclusions about the partitioning of the fusome during GSC divisions and the length of the GSC cell cycle. The live imaging in this study also helps to clarify controversies over the number and position of centrosomes in interphase GSCs.
Moreover, they provide strong evidence that centrosomes separate prior to duplication, as is seen in neuroblasts, rather than after duplication, as is seen in most cell types (including male GSCs). Lastly, they analyze spectrosome dynamics in mutants with tumorous GSC-like cells and conclude that divisions of GSC-like cells adjacent to the niche divide normally but those away from the niche divide symmetrically. Overall, this study provides detailed information that will be of broad use to the community and the conclusions are generally well-supported by the data. However, I would recommend that several points be addressed before publication.

Comments for the author

1. The observation that GSCs within the same niche can have very different spectrosome phase durations is very interesting. The authors should show an example of this in the figures or supplemental movies.
2. I found it difficult to see the par-1 signal in the nuclear space in Fig. 2a and Movie S2. Also, Movie S2 appears to end at NEP, not 20 minutes after NEP, as claimed on page 7.
3. The discussion of Fig. 5A in the text refers to average time points, rather than the specific timepoints shown in 5A, making the discussion in this section hard to follow.
4. The spectrosomes in Fig. 5B are hard to see, perhaps because they are not fully in focus. I would recommend showing the GFP channel separately or using an inset to highlight the spectrosome in this case. Likewise, the centrosomes are hard to see in Fig. 5A at t=9'.
5. In Fig. 8, an example of a mutant GSC that is adjacent to the niche and undergoes a stereotypic (asymmetric) division should be provided.
6. There are many grammatical errors throughout the text.

Reviewer 3

Advance summary and potential significance to field

Live Imaging of the Drosophila ovarian niche shows spectrosome and centrosome dynamics during asymmetric germline stem cell division by Villa-Fombuena et al.

Germline stem cells (GSCs) have been a long-standing important model for stem cell behavior and regulation. Here, the authors used an improved live-imaging technique to observe GSCs over extended time periods and to analyze the GSC divison pattern and behavior of organelles (spectrosomes and centrosomes/centrioles) in GSCs during the cell cycle in great detail.

The research described is largely descriptive but is important as it investigates the cell cycle related processes in GSCs in detail and very carefully in live ovaries. In addition, it clarifies several issues that were controversial in the field. This study was almost overdue - so, kudos to the authors. I believe that this paper will become a major reference in the field.

Comments for the author

The analysis has been conducted and documented very carefully in excellent figures, schematic drawings and movies. The manuscript is clearly written and carefully edited.

I have only one small critique point. The authors describe the spectrosomes as 'vesicular' in the Abstract.
This could lead to misunderstandings as spectrosomes (and fusomes) are not vesicles that are surrounded by a membrane, but instead contain membraneous material in form of vesicles or

© 2021. Published by The Company of Biologists under the terms of the Creative Commons Attribution License (https://creativecommons.org/licenses/by/4.0/).

tubular structures. A better description of the spectrosome organization in the introduction would be helpful.

Minor points:
Schematic drawings in Fig 1 and 5 overall are excellent. The authors might consider giving the cap cells and spectrosomes different colours to distinguish them clearly (in particular as they are in very close proximity. Also, it would also be helpful if the cap cells and spectrosome would be labeled in the top schematic of Fig 5.
Typos:
- p16 - 2nd last row: 'actins' -> actin
- p 18: 'fly's central nervous system' -> fly central ....
- p19 - 3rd last row: '...thought that are vulnerable ....' -> thought that they are vulnerable'
- p 20: KpnI and NotI -> first 3 letters in 'italics'
-p 21 -1st row: 'to the MatTek plate's bottom' -> to the bottom of the MatTek plate p 23: use 'nos'or 'nanos' consistently all figure legends: 10µm -> 10 µm

---

First revision

Author response to reviewers' comments

Reviewer 1: The authors have used live imaging of ex vivo cultured germaria to characterize the dynamics of GSC division. By using transgenic reporters that label components important for cell division, Villa-Fombuena and colleagues quantify duration of cell cycle phases, correlate spectrosome morphology with GSC cell cycle phase, examine GSC centrosome behavior before mitosis, and assess division of GSC-like cells in germline tumors. While the spectrosome morphological changes during the cell cycle have been previously described, the work discussed in this manuscript advances the field by carefully detailing morphological and temporal spectrosome and centrosome dynamics during GSC division. The manuscript is well written and experiments have been carefully executed but the novelty and implications for GSC/stem cell behavior are modest.
We thank the reviewer for their help with the evaluation of this work and for useful comments.

Major issues
Lines 96-98: Live imaging of the germaria has been previously described (Morris and Spradling, 2011; Valencia-Exposito, et al., 2016). While the authors mention that they have “defined experimental conditions that allow prolonged observations of ex vivo cultured germaria”, this is not a major part of the main text; instead, it has been relegated to the methods and supplementary data. If development of these conditions is a major part of being able to live image spectrosome behavior, the authors should consider making this a component of the main text. In addition, the implications of the work might be more obvious if the authors highlight how this tool can be used to analyze other aspects of oogenesis.
In its present form, the MS is above the word limit set by Development, so the additions that we can make to the text are limited in length. Still, we have now extended the description of our method in the main text.

Lines 157-158: It is not clear if development of the long-term ex vivo imaging system included a test of various time intervals. If so, it is worth mentioning to further support using the 10- minute interval. If not, a test of shorter imaging time intervals might identify a time interval that allows more frequent sampling for better time resolution.
The reason for choosing 10-minute intervals was two-fold. First, to avoid bleaching of the samples, as each time that germaria are scanned, we take 30-40 z-planes and the laser is on for 40-50 seconds. Second, to prevent photodamage. As we wanted to image a given germarium for the longest possible time, we went for long time intervals. We have used 90’’ (the settings used in our shorter movies) and 5’ intervals too. The increase in time resolution in the latter timepoints was not worthy, particularly considering that, after 3-4 hours of imaging, the fluorescent signals dropped considerably and the information obtained from the imaged germaria did not vary

© 2021. Published by The Company of Biologists under the terms of the Creative Commons Attribution License (https://creativecommons.org/licenses/by/4.0/).

significantly from the ones taken every 10 minutes. Thus, we assumed the compromise of 10-minute intervals. This information has been added to the relevant Experimental Procedures section.

In many cases, the authors do not indicate what percentage of GSCs analyzed display the pattern described. For example: lines 180-183, lines 203-206, lines 250-251, line 341, line 352, lines 414-417,
GFP::Par-1 signal recovery happened in 100% of GSCs analysed (lines 180-183); the addition of new material to the GSC and CB sides of the plug was also observed in all dividing GSCs analysed (lines 203-206); the occurrence of "anchorless" divisions is already quantified in the text (4 out of 11 scored divisions; lines 250-251); the data referring to the events during GSC division (line 341) are plotted in Fig S4, now cited more clearly in the text; the anterior centrosomes remained adjacent to the anterior cortex, next to the spectrosome, in all cases analysed (line 352). To make our findings clearer, we have revised the text and modified it accordingly.
Finally, regarding the movement of the anterior and posterior centrosomes prior to mitosis, the reviewer is right in that we only show the "average" speed and position of centrosomes. We have added a new supplementary figure (Fig S5) with the raw data (speed and position) for each of the quantified centrosomes.

Lines 414-417: How often did GSCs deviate from this "canonical" pattern? Was there another pattern observed?
We hope that the new Fig S5 helps to clarify this point.

Regarding statistical analysis, no data presented were quantified in such a way that statistical analysis could be conducted. Therefore, the "Statistical Analysis" section of Materials and Methods should be renamed to more accurately match what is being described.
We are unsure what the Reviewer means with this comment. We are happy to provide them with the excels containing the raw data in case they would like to revise our statistical analyses.

Minor Issues
Line 75: Change the "or" in "serine-threonine kinase Par-1 or the membrane skeletal" to "and"
Done.

Lines 77-80: Add in the additional references
We believe we have cited the appropriate references in this section of the text. Please advise if the Reviewer thinks otherwise.

Lines 92-95: There are additional references for this. For example, Dpp overexpression data shown in Xie and Spradling, 1998
Added.

Line 99: "stablish" should be "establish"
Corrected.

Lines 119-121: Since the change in GFP signal is very subtle, quantification may be a good way to exemplify this.
Done. We have confirmed that NEP induces an increase of at least 100% in GFP signal inside the nucleus. See new Fig. S1C.

Line 123-124: Indicate how many germaria this corresponds to.
To 11 germaria. This information has been added to the text.

Line 143-144: Add references
We believe we have cited the appropriate reference in this sentence. Please advise if the Reviewer thinks otherwise.

Line 147: "begun" should be "began" or "begins"
Corrected.

Line 171: "publish" should be "published"

© 2021. Published by The Company of Biologists under the terms of the Creative Commons Attribution License (https://creativecommons.org/licenses/by/4.0/).

Corrected.

Lines 180-183: It would be nice to see a quantification of Par1::GFP nucleoplasm intensity over each timepoint.
In relation to our response to the above "lines 119-121" comment, in which we have measured the increase in GFP signal in the nucleoplasm of diving GSCs, we have also quantified the concomitant decrease of spectrosome signal (down to 50% of the t= -50 value; new Fig. S1C).

Line 211: Blob word choice – maybe mass, or globule, etc
Changed to globule.

Line 251: "Of the 23 GSCs" – over how many germaria?
11. See response to "lines 123-124" comment above.

Line 292: Instead of "black", "lacking label", or something to that effect
Since we have used the expression of the GFP and RFP markers to define the different GSC types in the FlyFUCCI experiments (referring to green, red or yellow cells), we prefer to stick to our original nomenclature and name GSCs expressing neither GFP nor RFP as black GSCs.

Line 384: Instead of "We could observed", "We observed" or "We could observe"
Corrected.

Lines 454-456: not sure what this part of the sentence is supposed to say
We have modified the text to try and make the sentence clearer.

Reviewer 2: In this study, the authors combined short and long-term ex vivo live imaging with fluorescently tagged proteins to characterize the cell cycle of Drosophila germline stem cells. Their observations provide strong confirmation of previous conclusions about the partitioning of the fusome during GSC divisions and the length of the GSC cell cycle. The live imaging in this study also helps to clarify controversies over the number and position of centrosomes in interphase GSCs. Moreover, they provide strong evidence that centrosomes separate prior to duplication, as is seen in neuroblasts, rather than after duplication, as is seen in most cell types (including male GSCs). Lastly, they analyze spectrosome dynamics in mutants with tumorous GSC-like cells and conclude that divisions of GSC-like cells adjacent to the niche divide normally but those away from the niche divide symmetrically. Overall, this study provides detailed information that will be of broad use to the community and the conclusions are generally well-supported by the data. However, I would recommend that several points be addressed before publication.
We are grateful to the Reviewer for their positive comments and for the appraisal of our work.

1. The observation that GSCs within the same niche can have very different spectrosome phase durations is very interesting. The authors should show an example of this in the figures or supplemental movies.
Done. We have added a new panel to Fig. 1 (1E) with two GSCs from the same germarium showing different lengths of the same spectrosome phase

2. I found it difficult to see the par-1 signal in the nuclear space in Fig. 2a and Movie S2. Also, Movie S2 appears to end at NEP, not 20 minutes after NEP, as claimed on page 7.
We have increased the contrast in Movie S2 and in Fig. 2A to try and make the nuclear signal clearer. Please see in addition our response to Reviewer 1 comment on lines 119-121.
As for the end of Movie S2, it stops 70.5 minutes after NEP. What we mention in the text that takes place some 20' after NEP is the recovery of the GFP::Par-1 signal of the round-G1 spectrosome.

3. The discussion of Fig. 5A in the text refers to average time points, rather than the specific timepoints shown in 5A, making the discussion in this section hard to follow.
The reviewer is right. The text refers to the average time values of the different events in a GSC division, while panels in Fig 5A are examples of just one of the GSCs used to calculate the averaged values. We have modified Fig. 5, the main text and the figure legend to try and convey a clearer message.

4. The spectrosomes in Fig. 5B are hard to see, perhaps because they are not fully in focus. I

© 2021. Published by The Company of Biologists under the terms of the Creative Commons Attribution License (https://creativecommons.org/licenses/by/4.0/).

would recommend showing the GFP channel separately or using an inset to highlight the spectrosome in this case. Likewise, the centrosomes are hard to see in Fig. 5A at t=9'.
The spectrosome in panels 5B are difficult to see because they are labelled with GFP::Par-1, which shuttles to the nucleus during mitosis. For the sake of clarity and following the suggestion of the reviewer, we have added an inset to show the initial localisation of the anterior centrosome and the spectrosome (t= -6). Regarding the centrosomes in panel 5A (t= 9'), we could add some arrows should the reviewer wish to, but we think they may not be needed, as the centrosomes are found at the ends of this metaphase spindle.

5. In Fig. 8, an example of a mutant GSC that is adjacent to the niche and undergoes a stereotypic (asymmetric) division should be provided.
We have added a new movie (S15) and a new panel to Fig. 8 (8B) showing the division of a GSC located next to the cap cells.

6. There are many grammatical errors throughout the text.
Hopefully these have been corrected in this revised version of the MS.

Reviewer 3: Germline stem cells (GSCs) have been a long-standing important model for stem cell behavior and regulation. Here, the authors used an improved live-imaging technique to observe GSCs over extended time periods and to analyze the GSC divison pattern and behavior of organelles (spectrosomes and centrosomes/centrioles) in GSCs during the cell cycle in great detail. The research described is largely descriptive but is important as it investigates the cell cycle related processes in GSCs in detail and very carefully in live ovaries. In addition, it clarifies several issues that ere controversial in the field. This study was almost overdue - so, kudos to the authors. I believe that this paper will become a major reference in the field. The analysis has been conducted and documented very carefully in excellent figures, schematic drawings and movies. The manuscript is clearly written and carefully edited.
We very much appreciate the kind words of the reviewer and their suggestions to improve our MS.

I have only one small critique point. The authors describe the spectrosomes as 'vesicular' in the Abstract. This could lead to misunderstandings as spectrosomes (and fusomes) are not vesicles that are surrounded by a membrane, but instead contain membraneous material in form of vesicles or tubular structures. A better description of the spectrosome organization in the introduction would be helpful.
We have edited the Abstract and Introduction to account for this comment.

Minor points:
Schematic drawings in Fig 1 and 5 overall are excellent. The authors might consider giving the cap cells and spectrosomes different colours to distinguish them clearly (in particular as they are in very close proximity. Also, it would also be helpful if the cap cells and spectrosome would be labeled in the top schematic of Fig 5.
We have changed the colour code for the spectrosomes in the drawings in figs 1 and 5. We have also labelled the CpCs and the spectrosome in fig. 5C.

Typos:
- p16 - 2nd last row: 'actins' -> actin
- p 18: 'fly's central nervous system' -> fly central ....
- p19 - 3rd last row: '...thought that are vulnerable ....' -> thought that they are vulnerable'
- p 20: KpnI and NotI -> first 3 letters in 'italics'

-p 21 -1st row: 'to the MatTek plate's bottom' -> to the bottom of the MatTek plate p 23: use 'nos'or 'nanos' consistently
all figure legends: 10µm -> 10 µm
All the listed typos have been corrected.

Second decision letter

© 2021. Published by The Company of Biologists under the terms of the Creative Commons Attribution License (https://creativecommons.org/licenses/by/4.0/).

MS ID#: DEVELOP/2021/199716

MS TITLE: Live imaging of the *Drosophila* ovarian niche shows spectrosome and centrosome dynamics during asymmetric germline stem cell division

AUTHORS: Gema Villa-Fombuena, Maria Lobo-Pecellin, Miriam Marin-Menguiano, Patricia Rojas-Rios, and Acaimo Gonzalez-Reyes
ARTICLE TYPE: Research Article

I am happy to tell you that your manuscript has been accepted for publication in Development, pending our standard ethics checks. When you send in your final files, please do consider the suggestions of Reviewer 1 correct a few formatting issues and provide a few instances of even more clarify to this excellent MS.

Reviewer 1

Advance summary and potential significance to field

The authors have used live imaging of ex vivo cultured germaria to characterize the dynamics of GSC division. By using transgenic reporters that label components important for cell division, Villa-Fombuena and colleagues quantify duration of cell cycle phases, correlate spectrosome morphology with GSC cell cycle phase, examine GSC centrosome behavior before mitosis, and assess division of GSC-like cells in germline tumors. While the spectrosome morphological changes during the cell cycle have been previously described, the work discussed in this manuscript advances the field by carefully detailing morphological and temporal spectrosome and centrosome dynamics during GSC division. The manuscript is well written and experiments have been carefully executed. This revised version is suitable for publication.

Comments for the author

The authors seemed to have sufficiently addressed my comments and made appropriate revisions. Unfortunately, I do not have access to the response to reviewers document and am only able to notice some of the revisions. However, the manuscript in its current form is acceptable for publication.

Reviewer 2

Advance summary and potential significance to field

See my previous review.

Comments for the author

With this revised manuscript, the authors have addressed my previous concerns and I now fully support its publication in Development.

Reviewer 3

Advance summary and potential significance to field

see my previous review regarding significance.

Revised manuscript: Strong and improved version. Movies are excellent and very helpful.

Comments for the author

I only have some minor suggestions:

© 2021. Published by The Company of Biologists under the terms of the Creative Commons Attribution License (https://creativecommons.org/licenses/by/4.0/).

1. Intro:
Paragraph (P)1: revise 2nd sentence. The GSC niche develops at the larval/pupal stage (not the adult stage).
P1 and beginning of P2: add references
Row (R)87: Dpp -> Dpp pathway R87,90,91: dpp -> Dpp (non-italics)

Results:
- Whole manuscript and figure/movie legends: replace ‘fusion’ with ‘fusion protein’ (e.g. ‘GFP::Par1 fusion’
on R113), or delete ‘fusion’, or replace ‘fusion’ with ‘marker’.
- R119: not sure why the authors describe the signal of GFP::Par-1 as 'background signal'. Whatever signal there is should be real signal from GFP::Par-1 protein.
-R268/269: revise sentence e.g. to: We expressed these markers, which localize to the nucleus, with the help of ....
-R271: replace 'photodamage' with 'laser damage'?
-R271/272: revise sentence: Z-sections were collected at 1 µm intervals and Z-stacks were recorded every 10 minutes.
-R354: GSC's -> GScs
-R41-412: use the singular form for anterior and posterior centrosome in these sentences
-R441: What happens to the midbody in GSC tumours?

Discussion:
-R456: cap cells rosette -> cap cell rosette
-R505: Neuroblasts are stem cells! (not 'stem cell-like precursors')
-R517/518: replace 'statement' with e.g.. process or question
-R536: "Considering ...., a CSB divides ....." -> This suggests that on average a CSB divides ...
M&M:
566: fusion -> construct 584: Kcl -> KCl 589: NaCOH3 0.1 M -> 0.1 M NaCOH3 602: anti-hts -> anti-Hts
figure legends:
916: 'correspond to the mRFP::CycB and GFP::E2f1 fusions' -> correspond to mRFP::CycB and GFP::E2f1 respectively.
Fig 6: I suggest to add a more detailed description of the figure panels.
949: '3D rendition from' -> '3D rendition of
Figures:
Fig5A/B: provide full names: e,g.: 'condens' -> condensation
Fig 7: um -> µm
Suppl legends:
MovS9, R3: 'spectrosome undergoing cytokinesis ??? - revise
FigS5: R6'set' -> sets R9: 'in each' -> at each
MovS3, R3: cytoplasms -> cytoplasm

© 2021. Published by The Company of Biologists under the terms of the Creative Commons Attribution License (https://creativecommons.org/licenses/by/4.0/).
